# TCR repertoire of human cytotoxic CD4 T cells responding to betaherpesviruses HHV-6B and HCMV

**DOI:** 10.3389/fimmu.2025.1631558

**Published:** 2025-11-25

**Authors:** Aniuska Becerra-Artiles, Grant C. Weaver, Peter O. Oluoch, Lawrence J. Stern

**Affiliations:** 1 Department of Pathology, UMass Chan Medical School, Worcester, MA, United States; 2 Department of Microbiology, UMass Chan Medical School, Worcester, MA, United States; 3 Department of Biochemistry and Molecular Biotechnology, UMass Chan Medical School, Worcester, MA, United States

**Keywords:** cytotoxic CD4 T cells, TCR repertoire, public TCRs, HHV-6B, HCMV

## Abstract

**Background:**

The role of CD4 T cells in the control of viral infections beyond their traditional helper activity has been increasingly recognized, and CD4 T cells with cytotoxic capacity have been reported for the nearly ubiquitous betaherpesviruses HCMV (human cytomegalovirus) and HHV-6B (human herpesvirus 6B).

**Objective:**

We sought to investigate the functional landscape of cytotoxic CD4 T cells responding to HHV-6B and HCMV epitopes presented by DRB1*03:01 and to identify public T-cell receptors (TCRs) (i.e., shared by multiple subjects).

**Approach:**

We tetramer-sorted epitope-specific CD4 T cells from healthy donors and performed RNA and TCR sequencing to assess functional profiles and identify TCR clonotypes. We evaluated the publicity of the repertoire and tested the functionality, epitope specificity, and sensitivity of selected public clonotypes.

**Results:**

Differential gene expression analysis comparing T cells expanded with HHV-6B and HCMV epitopes showed differences in their functional profiles, with the HCMV-expanded T cells displaying a more robust cytotoxic gene expression signature. Tens to hundreds of TCR clonotypes responding to HHV-6B or HCMV were identified in each subject. The TCR repertoires were dominated by private clonotypes in all subjects, but 3 public TCRα/β, along with 41 public TCRα and TCRβ clonotypes were identified. Some of these clonotypes and closely related variants were found in a substantial fraction of DRB1*03:01 subjects in datasets of total peripheral blood TCR repertoires. TCRs associated with two HHV-6B epitopes (U11.306–323 and U85.88-104) and one HCMV epitope (pp65.509-523) were cloned for validation and biochemical characterization. Using an *in vitro* activation assay, the epitope specificity was confirmed for each selected TCRα/β, with half-maximal activation observed at 5–50 nM peptide concentration. With one exception, all TCRs bound tightly to the corresponding peptide-major histocompatibility complex (pMHC) tetramer. Finally, minimal peptide mapping combined with structural modeling of pMHCs identified potential sites of TCR interaction.

**Conclusions:**

CD4 T cells recognizing HHV-6B or HCMV exhibit cytotoxic signatures and can lyse antigen-pulsed target cells, with the HCMV-specific population exhibiting greater activity. The TCR repertoires of CD4 T cells recognizing HHV-6B or HCMV epitopes presented by DRB1*03:01 are broad but include public TCR clonotypes. These TCRs may be useful to monitor infection, reactivation under immunosuppressive conditions, and response to therapy.

## 1 Introduction

T cells are major players in the adaptive immune response that recognize peptide antigens presented on the surface of cells by major histocompatibility complexes (MHCs), with CD4 T cells recognizing antigens presented by class II MHC (MHC-II) and CD8 T cells recognizing peptides presented by class I MHC (MHC-I). The recognition is mediated by the T-cell receptor (TCR), a heterodimer expressed on the surface of T cells. The heterodimer components, the TCRα and TCRβ subunits, are encoded by the TRA and TRB genes and are produced during T-cell development by the somatic recombination of the variable (V), diversity (D), and joining (J) gene segments, combined with the introduction or deletion of nucleotides at the junctions. The hypervariable CDR3 region of the TCRs, which contains the VJ (TRA) or VDJ (TRB) junctions, interacts directly with the antigenic peptides and is responsible for much of the peptide-MHC (pMHC) specificity (1). The somatic recombination produces a pool of diverse T cells in the thymus, which, after positive and negative selection, constitutes the naïve T cells’ repertoire.

The high diversity of the TCR repertoire has been associated with the effectiveness of the T cells to recognize and respond to the countless epitopes that individuals may encounter throughout life (2, 3). The composition of the TCR repertoire reflects the antigenic exposure history of an individual (4–7). Diverse TCR repertoires can be involved in both beneficial and deleterious outcomes, including the control of infections, viral escape, autoimmunity, and control of cancers (8–10). Antigen-specific TCR repertoires are mostly distinct among individuals (i.e., private), but some TCRs are observed in multiple individuals and are referred to as public [reviewed in (2, 8)]. Public TCRs may arise in the pre-selection repertoire by (a) convergent recombination, where different recombination events lead to the same nucleotide sequence; (b) convergence of different nucleotide sequences coding for the same amino acid sequence; and (c) bias during the recombination, which included preferential gene usage, VDJ pairing, and nucleotide deletions/additions [reviewed in (2, 8, 11)]. These mechanisms occur regardless of the individuals’ HLAs. There has been interest in identifying shared or public TCR and in assessing the convergence of TCR repertoires within and among individuals, as shared repertoires may represent subsets of TCR that are effective in controlling an infection or involved in a pathologic response (12, 13). The usefulness of shared TCR repertoires in assigning antigen specificity as diagnostic tools or for HLA typing has also been explored (14, 15). Traditionally, studies describing TCR in infectious diseases, autoimmunity, and cancer focused on CD8 TCR repertoires and on the more variable TCRβ subunit. Next-generation sequencing and single-cell technology have facilitated TCR repertoire analysis and expanded beyond the traditional targets, including reporting of full TCRα/β clonotypes.

Human herpesviruses are large-genome double-stranded DNA viruses that establish lifelong latent infections after the primary encounter [reviewed in (16)]. Viral reactivation can occur in immunocompromised individuals and poses a risk for unwanted outcomes, including disease progression, birth defects, and transplant rejection [reviewed in (17, 18)]. Human cytomegalovirus (HHV-5 or HCMV) and human herpesvirus 6B (HHV-6B) are related betaherpesviruses with similar life cycles, genome organizations, and restricted host tropism (19). HCMV is highly prevalent worldwide, with a seroprevalence of 40%–70% in industrialized countries (20) and HHV-6B is almost ubiquitous, with a seroprevalence of 70%–100% (21). Both HCMV and HHV-6B have a wide cell tropism; for HCMV, the main replication sites are fibroblasts and epithelial, endothelial, and smooth muscle cells, while for HHV-6B, the preferred targets are CD4^+^ T cells but it can also infect other cells either productively or non-productively (22–26).

The cellular immune response to HCMV has been extensively studied [reviewed in (27)]. The total HCMV-specific T-cell responses in seropositive subjects can reach ∼10% of both the CD4^+^ and CD8^+^ memory compartments in peripheral blood (28). HCMV-specific CD8 T cells are a critical component of the immune response to the virus [reviewed in (29, 30)]. These cells are mostly categorized as effector memory cells (30). HCMV-specific CD4 T cells also have a protective role in HCMV immunity (29). CD4 helper T cells are essential for the effective CD8 T-cell responses (31), and a direct effector role of the HCMV-specific CD4 T cells in the control of the infection has also been suggested (32). In some cases, cell types that can be infected by HCMV have been shown to express MHC-II in physiologically relevant scenarios, suggesting a potential role for direct CD4^+^ T-cell recognition of infected cells. The cytotoxic activity of CD4 T cells against cells displaying HCMV-derived peptides on MHC-II has been reported (33), and upregulation of MHC-II on endothelial cells (34) and the presentation of peptides derived from HCMV glycoprotein gB on MHC-II on HCMV-infected epithelial cells and senescent fibroblasts have been described (35, 36). In fact, HCMV down-regulates MHC-II expression (37, 38), presumably as an immune evasion mechanism, which highlights the importance of CD4 T cells in the control of the virus.

Characterization of the T-cell response to HHV-6 has been more challenging. Persistent HHV-6 DNA in the blood of patients with lymphocytopenia and decreased proliferative responses suggest an important role of T cells in HHV-6 control (39). HHV-6B primary infection induces T-cell responses that peak later than other herpesviruses (40), possibly because of the tropism of the virus towards T cells, which could also affect the cellular immune response to other viruses (39). Both CD4 and CD8 T cells can proliferate in response to HHV-6 antigens (41), although a bias to CD4 T cells has been observed (39, 42, 43). In peripheral blood of healthy individuals, the T cells responding to HHV-6B are low frequency but can be expanded by *in vitro* stimulation with different antigens (43–49). T cells responding to HHV-6B antigens have been described as polyfunctional memory cells, which mainly produce interferon γ (IFNγ) and tumor necrosis factor α (TNFα), in addition to having cytotoxic capacity and activity (42–45, 48). CD4 T cells, the major target of HHV-6B infection, are known to express MHC-II molecules in response to IFNγ as part of inflammatory responses, including responses to virus infections (50). Early HHV-6 studies reported that a proportion of infected T cells expressed MHC DR antigen (51). This evidence indicates the possibility of a direct recognition of HHV-6B-infected CD4 T cells by cytotoxic CD4 T cells.

To our knowledge, an HHV-6B-specific TCR repertoire has not been reported yet. In contrast, several HCMV TCR repertoire studies are available for both CD8 and CD4 T cells (13, 52–61). In this work, we studied the CD4 T cells recognizing previously identified HHV-6B (48) and HCMV (62, 63) epitopes presented by HLA-DRB1*03:01. We expanded *in vitro* CD4 T cells from subjects with a partial HLA match using epitope peptides and sorted the specific cells with the corresponding tetramers. Tetramer-sorted cells, as well as unexpanded total CD4^+^ T cells from the same subjects, were used for both bulk and single-cell TCR and RNA sequencing. Gene expression analysis of the virus-specific CD4 T cells revealed an increased expression of genes involved in cytotoxic responses as compared to total resting CD4 T cells. The HCMV-specific population showed a more robust cytotoxic gene expression signature as compared to the HHV-6B-specific population. The TCR repertoires specific for HHV-6B and HCMV consisted of clonal T-cell populations, with some clonotypes highly expanded. Within the total repertoires, we identified 3 public TCRα/β pairs (1 HHV-6B and 2 HCMV-specific), 13 TCRα and 7 TCRβ HHV-6B-specific clonotypes, and 13 TCRα and 9 TCRβ HCMV-specific clonotypes, shared in two or more subjects. Some public TCRs were more frequently observed in DRB1*03:01-positive subjects in other TCR datasets from total unsorted cells. Selected TCRα/β pairs were cloned, expressed in TCR-deficient T-cell lines, and used to validate antigen specificity, peptide sensitivity, and tetramer avidity and likely explore pMHC–TCR contacts.

## 2 Materials and methods

### 2.1 Study subjects and sample collection

Whole blood from nine subjects expressing HLA-DRB1*03:01 was collected under a protocol approved by the UMass Chan Medical School Institutional Review Board, and informed consent was obtained from all subjects. Peripheral blood mononuclear cells (PBMCs) were isolated using Ficoll-Paque (Cytiva, Marlborough, MA) density gradient centrifugation and used fresh or cryopreserved until use. HLA typing was performed at UMass Memorial Histocompatibility Laboratory.

### 2.2 Antigen-specific T-cell expansion

Antigen-specific T-cell lines were expanded by *in vitro* stimulation of fresh or frozen PBMCs with a pool of six HHV-6B peptides, individual HHV-6B peptides, or HCMV pp65.509–523 peptides (**Table 1**). Peptides were added at 10 μg/mL and cultures were maintained in RPMI 1640 supplemented with 10% AB+ human serum (Interstate Blood Bank, Inc., Memphis, TN), 50 μM beta-mercaptoethanol, 1 mM non-essential amino acids, 1 mM sodium pyruvate (all Gibco, Grand Island, NY), 100 U/mL penicillin, and 100 mg/mL streptomycin. On days 4 and 8, IL-2 (100 U/mL; Chiron, Emeryville, CA) was added and cells were maintained for ~12–15 days.

**Table 1:** Peptides used in the study.

Virus	Source protein^1^	Alias^2^	Location^3^	Sequence^4^	HLA^5^
HHV-6B	Large structural phosphoprotein	U11	308–323	SLPSISIDTKRPSADL	B1*03:01
HHV-6B	Putative OX-2 membrane glycoprotein homolog	U85	088–104	KINNLDVDSKLYFHIKH	B1*03:01
HHV-6B	Triplex capsid protein 2	U56	176–193	IKLPDIVNDKQSMYSMKT	B1*03:01^a^
HHV-6B	Envelope glycoprotein H	U48	476–490	ASPVRRDVTNSFVKT	B1*03:01^b^
HHV-6B	Envelope glycoprotein B	U39	117–130	MPVYEANLVNSRAQ	B3*02:02
HHV-6B	Protein U63	U63	131–147	IIDSIIKDGKFIKNVED	B1*03:01
HCMV	65 kDa phosphoprotein	pp65	509–523	KYQEFFWDANDIYRI	B1*03:01^c^

^1^ UniProt name of the protein to which the peptide belongs.^2^ Short identification used in this work.^3^ First and last amino acid position for the peptides in the source protein.^4^ Amino acid sequence.^5^ Presenting HLA allele used in this work [^a^ This peptide was also observed to bind to DRB3*01:01 (48); ^b^ This peptide was also observed to bind to DRB3*01:01, DRB1*13:01, and DRB3*02:02 (48); ^c^ No direct MHC–peptide binding data has been reported for this peptide. The peptide was previously reported to induce proliferation in cells from DR3+ (DRB1*03:01/DRB3*01:01) subjects (62) and the DRB1*03:01 tetramer recognized T cells from DR3+ subjects stimulated *in vitro* with the peptide (63)].

### 2.3 T-cell cytotoxicity assay

The capacity of expanded CD4 T cells to kill specific targets was assessed as described before (48). Briefly, we used the DRB1*03:01 cell line SupT1.CIITA (64) [kindly provided by Dr. Karen Duus (Touro University, Henderson, NV)] as target cells. These cells were maintained in RPMI 1640 medium supplemented with L-glutamine (2 mM), penicillin (100 U/mL), streptomycin (100 µg/mL) and 10% fetal bovine serum (FBS). Target cells were labeled with carboxyfluorescein succinimidyl ester (CFSE; Cell trace CFSE, Invitrogen, Carlsbad, CA) at 3 µM (CFSEhigh) or 0.35 µM (CFSElow) and loaded with an HHV-6B pool of peptides or the HCMV peptide (CFSEhigh), or a pool of self-peptides as control (CFSElow) for 1 h. Cells were washed and mixed 1:1 (high:low for each virus) and incubated overnight with T cells. Cells were collected, washed, and stained for dead cells (Live/Dead Fixable Violet Dead Cell Stain Kit™, Thermo Fisher Scientific, Waltham, MA) and HLA-DR (clone G46-6; BD Biosciences, San Jose, CA). Data were acquired using a BD LRSII flow cytometer equipped with BD FACSDiva software (BD Biosciences, San Jose, CA) and analyzed using FlowJo v. 10.7.1 (FlowJo, LLC, Ashland, OR). Gating was set to select total CFSE^+^ cells, HLA-DR^+^ cells, and live cells. The numbers of CFSEhigh and CFSElow cells in the live gate were determined for each sample and used to calculate the percentage of specific lysis. The pool of self-peptides consisted of eight abundant naturally MHC-II presented peptides: IEKFEKEAAEMGKGSF (Elongation factor 1-alpha 1), APSTYAHLSPAKTPPPPA (Lipolysis-stimulated lipoprotein receptor), KTTIRLMNSQLVTTEK (Beta-galactoside-alpha-2,6-sialyltransferase 1), KPVSKMRMATPLLMQ (HLA class II histocompatibility antigen gamma chain), RVEYHFLSPYVSPKE (Transferrin receptor protein 1), TGSWIGLRNLDLKGEF (Low-affinity immunoglobulin epsilon Fc receptor), LATWTIQGAANALSGD (Transferrin receptor protein 1), and SNRVVDLMAHMASKE (Glyceraldehyde-3-phosphate dehydrogenase).

### 2.4 Cell staining and tetramer sorting

PE- and APC-conjugated tetramers for each peptide (**Table 1**) were obtained from the NIH Tetramer Core Facility (Emory University, Atlanta, GA). PBMCs or expanded T cells were collected and washed, and dead cells were stained with Live/Dead Fixable Aqua Dead Cell Stain Kit™ (Thermo Fisher Scientific, Waltham, MA); Fc receptors were blocked with human Ig (Sigma-Aldrich, St. Louis, MO). Tetramers were added as a mix of PE and APC (final concentration of 4 µg/mL each) and staining was performed at 37°C for 2 h (CLIP tetramers were used as negative controls). Surface staining with antibodies CD3-APC-H7, CD4-PerCP-Cy5.5, CD8-APC-R700, CD14-BV510, CD19-BV510, CD56-BV510, CCR7-BV421, and CD45RA-BV620 was performed for the last 20 min of tetramer incubation, followed by washes, resuspension in buffer, and filtration through a 0.4-µm membrane. Data were acquired using a BD LRSII flow cytometer equipped with BD FACSDiva software (BD Biosciences, San Jose, CA) and analyzed using FlowJo v. 10.7.1 (FlowJo, LLC, Ashland, OR). The gating strategy consisted of selecting lymphocytes and single cells, discarding cells in the dump channel (dead, CD14^+^, CD19^+^, and CD56^+^ cells), selecting CD3^+^CD4^+^CD8^−^ cells, and assessing the tetramer double-staining PE^+^APC^+^ in this population. The CD45RA/CCR7 staining was assessed in both the CD3^+^CD4^+^CD8^−^ and PE^+^APC^+^ populations. For sorting, we used the BD FACS Aria Cell Sorter at The UMASS Flow Cytometry Core Facility. For *in vitro* expanded cell populations, cells in the PE^+^APC^+^ gate were sorted. For resting unexpanded CD4 T populations, cells in the CD3^+^CD4^+^CD8^−^ gate were collected. Sorted cells were washed and used immediately in the 10X Genomics single-cell VDJ pipeline or used to prepare total RNA (RNeasy Mini or RNeasy Micro kits, QIAGEN, Germantown, MD) for bulk sequencing.

### 2.5 Bulk TCR sequencing

Bulk TCR sequencing was done as described before (65), using an in-house template-switching 5′-RACE (Rapid Amplification of cDNA Ends) approach adapted (66) to amplify and tag TCR mRNAs (67). Briefly, total RNA with an RQN above 7.2 (Bioanalyzer Service at UMASS Molecular Biology Core Labs) was used for library preparation. RNA (~100 ng) was annealed for 3 min at 72°C with 1 µM primers recognizing TRAC or TRBC. All primers and oligos were obtained from Integrated DNA Technologies (IDT, Coralville, IA); sequences are shown in **Supplementary Table S1**. Reverse transcription mixture containing 1 µM UMI/R1 oligo, 5 U/µL SMARTScribe reverse transcriptase, 0.5 mM dNTP, 2 U/µL RNase inhibitor (all Takara Bio USA, Inc, Mountain View, CA), 5 mM DTT, 1 M betaine, and 6 mM MgCl_2_ (all Invitrogen, Thermo Fisher Scientific) was added and incubated at 42°C for 90 min followed by 10 cycles of 50°C/42°C for 2 min each, with final incubation at 70°C for 15 min. Excess oligo was removed by incubating at 37°C for 40 min with 214 U/mL Uracil DNA glycosylase (New England Biolabs, Ipswich, MA). cDNA was purified using AMPure XP Beads (Beckman Coulter, Brea, CA) following the manufacturer’s instructions. Four PCR reactions were performed to add R2, P5, P7, and i7 indices to the transcripts. All reactions were performed at a final concentration of 0.2 µM primers, 0.02 U/µL KOD Hot Start DNA Polymerase, 0.2 mM dNTP, and 1.5 mM MgSO_4_ (all Novagen/Millipore Sigma, Burlington, MA). The first PCR utilizes purified cDNA and second-strand and RT8 primers; the second PCR utilizes the purified product from the previous PCR and second-strand and nested primers; the third PCR utilizes the purified product from the second PCR and 5′RACE and barcodes/i7 index primers; the fourth PCR utilizes the purified product from the third PCR and P1 and P2 primers. Cycling conditions for PCR1 were as follows: 95°C for 2 min; 10 cycles of 95°C for 20 s, 70°C for 10 s (−1°C per cycle), 70°C for 30 s; 15 cycles of 95°C for 20 s, 60°C for 10 s, 70°C for 30 s; final extension at 70°C for 3.5 min. Cycling conditions for PCR2 and PCR3 were as follows: 95°C for 2 min; 8 cycles of 95°C for 20 s, 60°C for 10 s, 70°C for 30 s; final extension at 70°C for 3.5 min. Cycling conditions for PCR4 were as follows: 95°C for 2 min; 7 cycles of 95°C for 20 s, 60°C for 10 s, 70°C for 30 s; final extension at 70°C for 3.5 min. PCR products from PCR1–3 were purified using AMPure XP magnetic beads, and the final PCR product was purified using the QIAquick Gel Extraction kit (QIAGEN, Germantown, MD). TRA and TRB libraries were quantified (Fragment Analyzer Service at UMASS Molecular Biology Core Labs). Equimolar concentrations of 8–12 libraries were mixed and sequenced in an Illumina MiSeq System (paired-end reads, 250 cycles; UMASS Deep Sequencing Core Facility).

### 2.6 RNA sequencing

Bulk RNA sequencing was performed on 250 ng of RNA obtained as described in the previous section using the Illumina TruSeqv2 platform following the manufacturer’s instructions. Briefly, mRNA was isolated using poly dT beads, followed by fragmentation, cDNA synthesis using random hexamers, A-tailing and ligation of sequences for R1 and R2 primers, P5 and P7 adaptors, and i7 indices. Libraries were quantified using the Bioanalyzer, multiplexed, and analyzed in a single lane in the HiSeq4000 System (single read, 100 cycles; UMASS Deep Sequencing Core Facility).

### 2.7 Single-cell sequencing

For single-cell sequencing, the Chromium Single Cell VDJ v2 kit (10X Genomics, Pleasanton, CA) was used. Targeted cells were between 4,000 (3.1% multiplets) and 10,000 (7.6% multiplets). TCR and gene expression libraries were prepared following the manufacturer’s instructions. Libraries were quantified using qPCR (Kappa Library Quantification kit, Roche, Wilmington, MA), and quality control was performed using the Fragment Analyzer Service at UMASS Molecular Biology Core Labs. Sequencing was performed in the Illumina HiSeq4000 System (paired-end reads 26 × 96 cycles; UMASS Deep Sequencing Core Facility).

### 2.8 Differential gene expression analysis

For bulk data, demultiplexed raw data were processed using the DoplhinNext RNASeq pipeline [Biocore DolphinNext (68)]. Differential expression (DE) was performed using the DESeq2 algorithm in DEBrowser v1.15.2. Significant results were selected using *p*_adj_ < 0.05 and fold change > 5. Biological replicates for total CD4 T cells (three subjects), HHV-6B-expanded (three subjects), and HCMV-expanded (two subjects) were used for the analysis.

Single-cell data were analyzed using the 10X Genomics Cell Ranger 3.1.0 and Loupe Cell Browser 3.1.1 pipelines. Biological replicates for total unstimulated CD4 T cells, HHV-6B-expanded, and HCMV-expanded (two subjects each) were used for the analysis. Results from different samples were combined (using the Aggregate algorithm), and data were visualized using UMAP (Uniform Manifold Approximation and Projection) for data reduction and clustering in the Loupe Cell browser application, using *K*-means = 5.

Overrepresentation analysis (ORA) or gene set enrichment analysis was performed using g:Profiler [gSCS threshold (69)]. Differentially upregulated genes with *p*-values < 0.05 and more than two- or fivefold change (single-cell and bulk, respectively) were used as input lists in the analyses. Results were visualized using Cytoscape v3.10; enrichment maps of networks are presented [node fill color = −log_10_(*p*-value)].

### 2.9 TCR repertoire analysis

TCR libraries were demultiplexed, error-corrected, counted, and assigned to TRAV/TRAJ or TRBV/TRBJ genes using MiGEC v1.2.9 and MiXCR v3.0.13 pipelines (70, 71). The quality scores for selected CDR3 sequences were >21 (99% accuracy in base call). The output of MiXCR was used for further data analysis with VDJTools v1.2.1 (72) for repertoire statistics, diversity, and clonality measurements; GLIPH2 (12) and TCRDist (73) were used for the clustering and identification of patterns and motifs.

To find CDR3α or CDR3β public sequences, we used the Python library Pandas (v1.5.2). Datasets for each subject (bulk and uncoupled single-cell data, when available) were combined and reduced to unique CDR3. Datasets from all subjects for a condition (HHV-6B, HCMV, and CD4) were combined and used to find identical sequences shared by two or more subjects. To find public sequences in published datasets available online, CDR3β sequences were obtained from the databases listed below, and the data were reduced to unique sequences per subject and combined with our data for analysis. The publicly available datasets include the following: CDR3β sequences derived from unsorted PBMCs or total CD4^+^ cells from 5 subjects reported by Soto et al. (74) and from 63 subjects reported by Nolan et al. (75); productive rearrangements were downloaded from the immuneACCESS portal (https://doi.org/10.21417/CS2020CR and https://doi.org/10.21417/ADPT2020COVID, respectively; both datasets combined represented almost 15,000,000 sequences, and all the subjects selected had HLA typing information available. For antigen-specific data, we compiled a list of CDR3β sequences using the following sources: 164 CDR3β sequences associated to HCMV reported by Emerson et al. [ (14); 45 sequences were restricted to MHC-I, and for the rest, there was no restriction information]; 917 previously reported CDR3β sequences recognizing HCMV antigens also used by Emerson et al. in their analysis (these TCRs were ~90% restricted to MHC-I); 1,452 HLA-restricted and antigen-specific CDR3β sequences (for IAV, HCMV, HIV, SARS-CoV-2, *Mycobacterium tuberculosis*, and *Escherichia coli*) downloaded from VDJdb for the HLA alleles DRB1*01, 04, 07, 15, and DRB1*03, DRB3*02, and 03 [ (76); https://vdjdb.cdr3.net/search]; 843 CDR3β from CD8 T cells of 6 subjects expanded *in vitro* and sorted with A*02:01-M1.58-66 (77); 166 CDR3β from PBMCs of 13 subjects sorted with A*02:01-pp65.495-503 (78); 382 CDR3β from CD4 T cells sorted with DPA1*01:03/DPB1*04:01-SARS-CoV-2 S.167-180 (79); 187 CDR3β sequences from T cells from 4 subjects expanded *in vitro* with spike protein from human common-cold coronavirus (HCoV) peptide pools and sorted with DPA1*01:03/DPB1*04:01-SARS-CoV-2 S.815-829 (65); 2,415 CDR3β from expanded but unsorted T cells from 7 subjects (65); and 885 CDR3β from T cells of 3 subjects expanded *in vitro* with a pp65 peptides pool and sorted using activation markers (60). Contingency analysis was used to calculate the odds ratio and significance.

### 2.10 TCR cloning and lentivirus production

CDR3α and CDR3β nucleotide sequences, and V, D, J, and C gene information were obtained from the single-cell data. *In silico* assembly of TRA and TRB genes was done by combining the CDR3 sequences with the V, D, J, and C genes, obtained from the IMGT database (https://www.imgt.org). The IL-2 signal sequence was added at the 5′ end of the construct, before the V gene; a stop codon was added at the end of the C gene; two cloning restriction sites were added flanking the whole construct (XbaI at the 5′ end and BamHI at the 3′ end). Each TCR chain was checked and edited for unwanted restriction sites. The TCR genes were obtained in pUC57 plasmids (GenScript USA Inc., Piscataway, NJ). The genes were cloned out of pUC57 using XbaI and BamHI restriction enzymes (New England Biolabs, Ipswich, MA), ligated into pCDH lentivectors carrying puromycin or IRES-GFP, and used to transform *E. coli* (NEB Turbo competent cells, New England Biolabs, Ipswich, MA). Positive colonies were expanded, plasmids were purified (QIAGEN Miniprep kit), and sequences were checked using Sanger sequencing (Azenta Life Sciences, Waltham, MA). Lentivirus was prepared in HEK293T cells [maintained in DMEM medium supplemented with 10% FBS, L-glutamine (2 mM), penicillin (100 U/mL), and streptomycin (100 µg/mL)], by adding a mix of pCDH, psPAX2, and pMD2.G vectors in a ratio of 3:2:1 in OptiMem medium (GIBCO, Thermo Fisher Scientific) plus PEI-Max (135 µg/mL; Polysciences, Inc., Warrington, PA) and incubating for 48 h. The lentivirus was concentrated from cell-free supernatants (100 kDa MWCO, Amicon ultrafiltration devices, MilliporeSigma, MA) and used to transduce Jurkat cells.

### 2.11 Production of single-TCR transduced cells

We used the Jurkat derivative cell line lacking expression of both TCR α and β subunits (J76.−/−) expressing CD4 (78, 80); this cell line was kindly provided by Dr. E. Mellins (Stanford University). J76.−/− were infected with one TCRα (pCDH-puromycin) and one TCRβ (pCDH-IRES-GFP) lentivirus in RPMI supplemented with 10% FBS, L-glutamine (2 mM), penicillin (100 U/mL), and streptomycin (100 µg/mL), in the presence of 10 µg/mL polybrene (MilliporeSigma, MA). In one case, a single construct carrying both TRB and TRA genes separated by a P2A sequence was used and introduced in pCDH-puromycin. After 72 h, the medium was replaced by RPMI + 10% FBS + 2 µg/mL puromycin and incubated for various days. TCRα/β expression was checked by flow cytometry using a monoclonal antibody that recognizes TCRα/β heterodimers (clone IP26, BioLegend Inc.), as well as GFP for TCRβ chain. TCRα/β-positive cells were sorted (UMass Chan Flow Cytometry Core Facility), expanded, and used for further experiments.

### 2.12 CD69 activation assay

Single TCRα/β transduced cells were co-cultured with the cell line Vavy (9063; DRB1*03:01/DRB3*01:01) pulsed with peptide at various concentrations for 15 h, after which cells were washed and stained for CD69 (clone FN50, BioLegend, Inc.) or TCRα/β (monoclonal antibody IP26) for 30 min. After washes, 7AAD was added and samples were filtered through a 0.7-µm membrane before data acquisition. Data were acquired using a BD LRSII or Symphony A5 flow cytometer equipped with BD FACSDiva software (BD Biosciences, San Jose, CA) and analyzed using FlowJo v. 10.7.1 (FlowJo, LLC, Ashland, OR). The gating strategy consisted of selecting single cells, followed by discarding dead cells, selecting TCRα/β+ cells, and assessing the CD69-APC levels in this population.

### 2.13 Modeling

AlphaFold2 (81, 82) was used to build preliminary pMHC models. DRA*01:01 and DRB1*03:01 (both from the IMGT website, https://www.ebi.ac.uk/ipd/imgt/hla/alleles) and peptides U11, U85, and pp65 were loaded and analyzed using multimer mode, as previously reported for MHC-I peptide complexes (83). For modeling of MHC-II peptide complexes, we computationally attached the peptide to the beta subunit using a GSGGSIEGRGGSGAS linker. The resulting models were inspected for the presence of the canonical MHC-II peptide hydrogen-bonding interactions (84). Models were visualized using the PyMOL Molecular Graphics System (85).

### 2.14 Statistical analysis

Graphs and statistical analyses were performed using GraphPad Prism 10.4.1 (GraphPad Software, San Diego, CA).

## 3 Results

### 3.1 Expansion and isolation of antigen-specific HHV-6B and HCMV CD4 T cells

**Figure 1:**
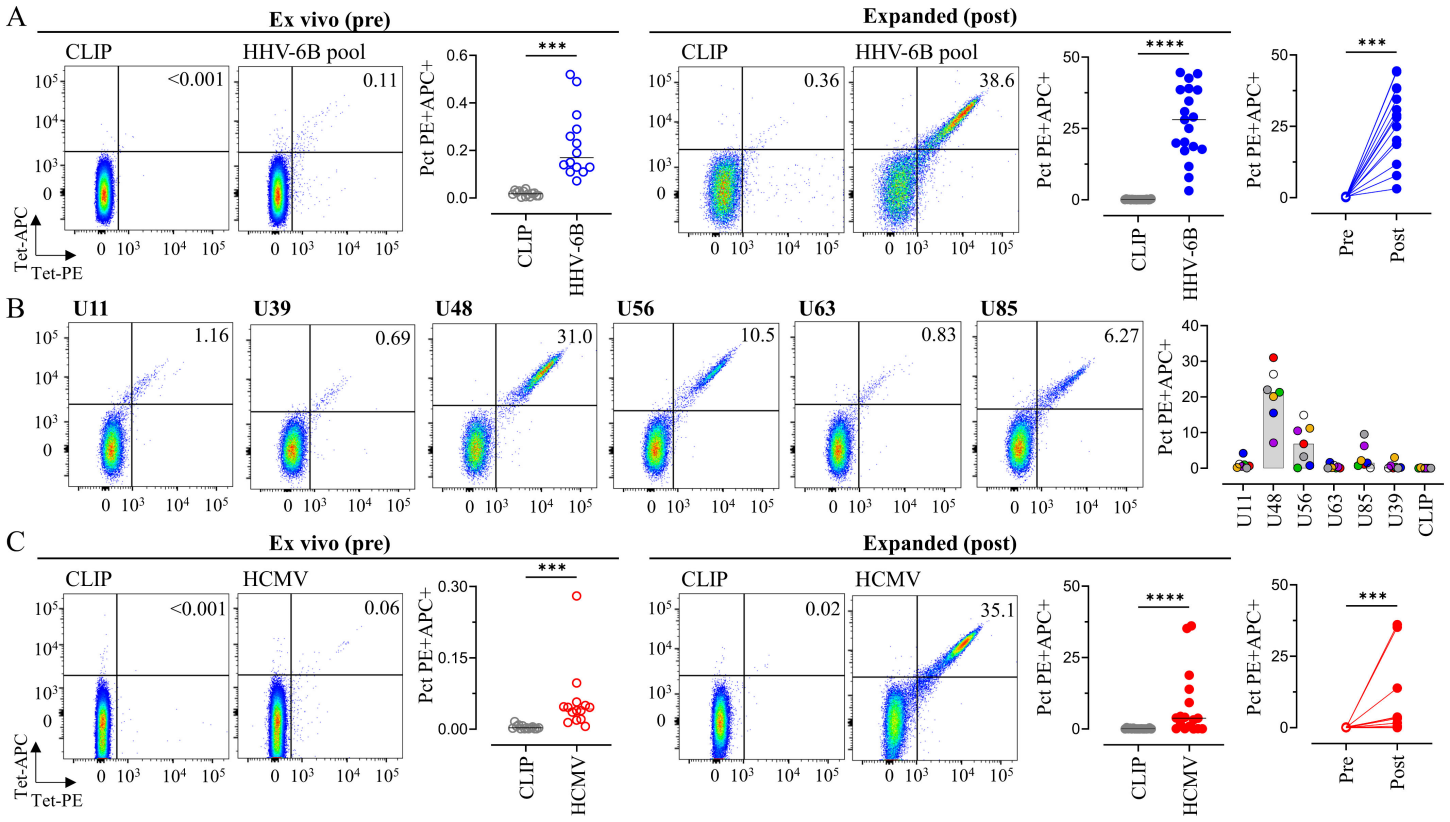
MHC tetramer staining of HHV-6B- and HCMV-specific T cells directly *ex vivo* and after *in vitro* expansion. **(A)** HHV-6B-specific T cells present in PBMC or after 14 days of stimulation with a mix of six antigenic peptides were detected using a mixture of the six HHV-6B tetramers (**Table 1**); as negative control, a mixture of the CLIP tetramers was used. Representative dot plots for double tetramer staining for one subject, summary of samples from nine subjects at each time point (14 *ex vivo*, 19 after expansion), and comparison pre/post expansion for paired samples (*n* = 14). **(B)** HHV-6B-specific T cells were detected by staining using individual tetramers in *in vitro* expanded cells; representative dot plots for each tetramer and summary of data obtained from seven subjects. **(C)** HCMV-specific T cells present in PBMC or after 14 days of stimulation with the pp65 peptide were detected using the single tetramer; representative dot plots for the double tetramer staining for one subject, summary of samples from nine subjects at each time point (14 *ex vivo*, 17 after expansion), and comparison pre/post expansion for paired samples (*n* = 14). In all cases, each tetramer was used as both PE and APC-conjugated, to improve gating on the positive populations; statistical analyses were done by paired non-parametric *t*-test (***p < 0.001, ****p < 0.0001). Empty symbols were used for pre-expansion and filled symbols were used for post-expansion conditions. The gating strategy is shown in **Supplementary Figure S1**.

T cells responding to HHV-6 are usually found at low frequencies in the blood of healthy subjects (44, 47). To facilitate the isolation of antigen-specific populations, we increased the frequency of antigen-specific T cells by *in vitro* expansion with a pool of six previously identified antigenic peptides from HHV-6B. These peptides were originally identified by mass spectrometry in the natural MHC-II immunopeptidome of HHV-6B-infected cells (48). The six-peptide pool was recognized by essentially all subjects with the common HLA-DR3 haplotype, consistent with the near-universal seroprevalence for HHV-6B [reviewed in (21)]. Most of the epitopes were presented by DRB1*03:01, and T-cell responses were mainly associated with this allele, except for U39.117-130, which was presented by DRB3*02:02 (**Table 1**). HHV-6B-specific T cells were expanded with the pool of the six epitope peptides and detected with a mixture of the corresponding six tetramers (**Figure 1A**). Across nine subjects tested, the average frequencies were 0.23% ± 0.14% *ex vivo* and 28.3% ± 11.3% after expansion, with all subjects showing significant expansion (**Figure 1A**). Responses to individual HHV-6B peptides were variable across subjects (**Figure 1B**) but expanded T cells of at least one of the peptides could be detected in all subjects studied. We also investigated a previously characterized HCMV-derived epitope from the 65-kDa phosphoprotein (pp65), presented by DRB1*03:01 (62, 63). The single pp65-derived peptide (**Table 1**) and tetramer were used for the expansion and detection of HCMV CD4 T cells (**Figure 1C**). Across the subjects tested, the average frequencies were 0.06% ± 0.08% directly *ex vivo* and 4.2% ± 6.7% after expansion. Significant expansion was observed only in samples from four subjects. We did not have serum available from these subjects for serological testing to assess which donors were seropositive for HCMV, although the fraction of subjects that expanded T cells after *in vivo* stimulation is consistent with the expected 50%–70% seroprevalence of HCMV infection (86). To determine whether the observed expansion represented antigen-specific populations present *ex vivo*, we assessed *ex vivo* tetramer staining frequencies for the subjects for whom expansion after *in vitro* stimulation was observed with those with no expansion. The four donors for whom expansion was observed had tetramer-staining frequencies in PBMCs significantly above background (0.070% ± 0.077% for the specific tetramer and 0.005% ± 0.005% for the CLIP tetramer background, *p* = 0.0039) as compared to the donors for whom expansion was not observed where specific staining was not observed (0.023% ± 0.011% for the specific tetramer and 0.003% ± 0.002% for the CLIP tetramer, *p* > 0.05). Overall, we observed relatively low frequencies of DR3-restricted CD4 T cells responding to HHV-6B and HCMV epitopes, which could be substantially expanded *in vitro*.

### 3.2 Phenotypic and functional characterization of antigen-expanded CD4 T cells

**Figure 2:**
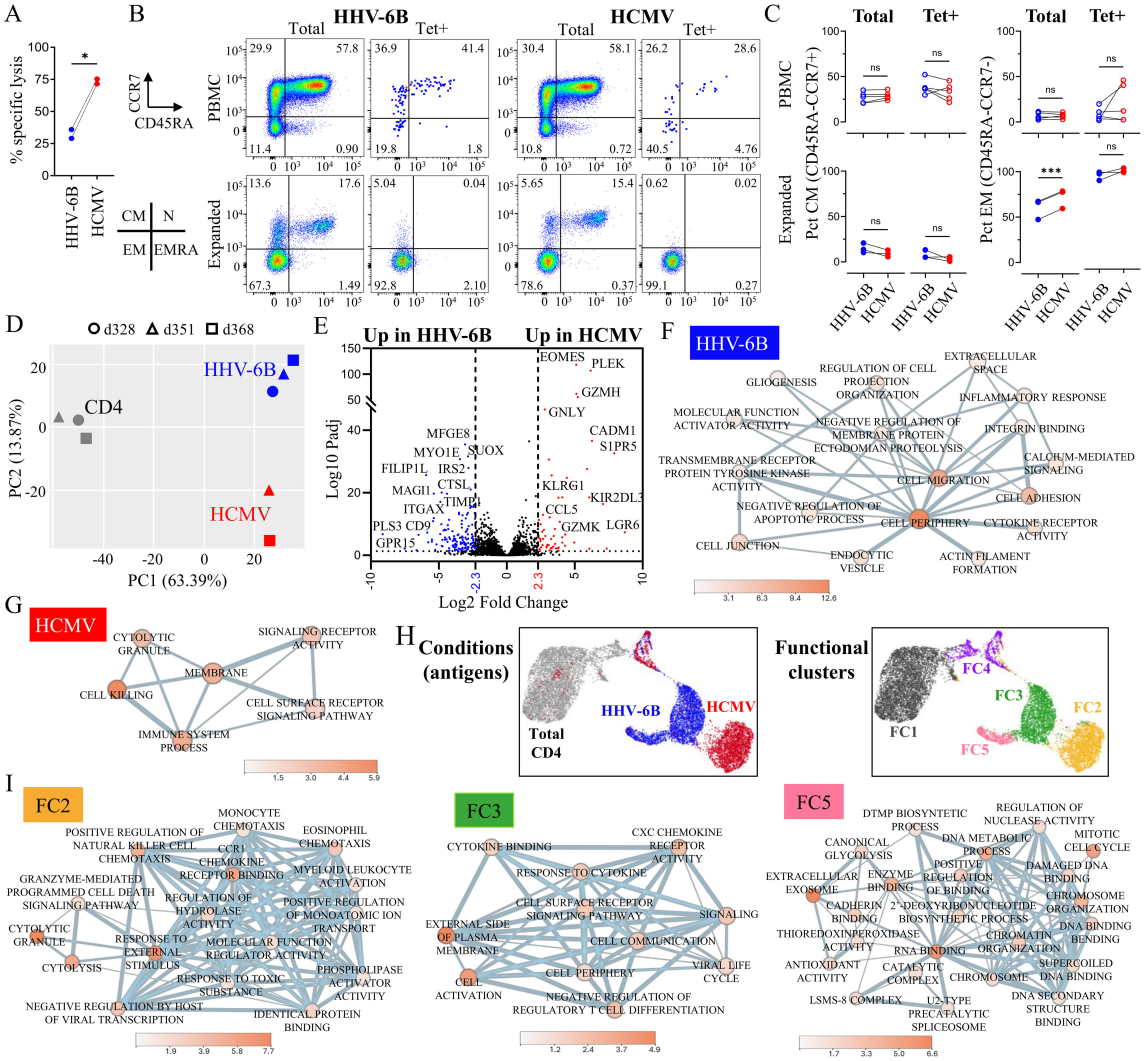
Functional characterization of HHV-6B- and HCMV-*in vitro* expanded T cells. **(A)** Cytolytic activity of antigen-specific expanded T cells; T cells from a subject were expanded in parallel with the pool of six HHV-6B peptides or the HCMV single peptide, and the specific lysis was measured after 14 days using target cells pulsed with the same antigens used for expansion and a target-to-effector (T/E) ratio of 1:5; statistical analysis by paired *t*-test (*p = 0.025). **(B)** Expression of CCR7 and CD45RA on total CD4 T cells (Total) or tetramer-positive CD4 T cells (Tet+), in unstimulated PBMC or after *in vitro* expansion; the memory subsets were defined as follows: naïve (N, CD45RA+CCR7+), central-memory (CM, CD45RA−CCR7+), effector-memory (EM, CD45RA−CCR7−), and EM re-expressing RA (EMRA, CD45RA+CCR7−); representative dot plots and summary for the CM and EM subsets. Statistical analysis by paired *t*-test (***p < 0.001; n.s., not significant). The gating strategy is shown in **Supplementary Figure S1**. **(C)** Comparison of the percentage of HHV-6B- and HCMV-expanded T cells (total or tetramer positive) that are in the CM or EM subsets; statistical analysis by paired *t*-test. **(D)** Principal component analysis (PCA) of the gene expression of HHV-6B-expanded/tetramer-sorted T cells (three subjects), HCMV-expanded/tetramer-sorted T cells (three subjects), and unexpanded total CD4 T cells (three subjects) obtained by bulk RNA sequencing. **(E)** Volcano plot representation of the differentially regulated genes between HHV-6B and HCMV samples; in color are shown the statistically significant genes upregulated at least fivefold in HHV-6B (blue) or HCMV (red), *p*_adj_ < 0.05. **(F)** Functional profile network for genes upregulated fivefold or more in HHV-6B-expanded cells compared to HCMV-expanded cells. **(G)** Functional profile network for genes upregulated fivefold or more in HCMV-expanded cells compared to HHV-6B-expanded cells. **(H)** Single-cell differential gene expression analysis on HHV-6B-expanded/tetramer-sorted, HCMV-expanded/tetramer-sorted, and unexpanded total CD4 T cells (UMAP plots, K-mean = 5, data from two subjects); clusters are colored by condition (HHV-6B, HCMV, or total CD4) or functional profile (FC1 to 5). **(I)** Functional profile network for genes upregulated threefold or more in cluster FC2, FC3, and FC5. For all networks, node fill color = −Log10(*p*-value).

In a previous work, we assessed the bulk population of HHV-6B-specific CD4 T cells expanded *in vitro* by intracellular cytokine staining and cytolytic functional assays, showing that the responding cells are polyfunctional and kill target cells presenting the specific epitopes (48). Recently, Fastenackels et al. showed phenotypic differences between HHV-6 and HCMV responding T cells (87). Here, we compared the relative cytolytic activity of HHV-6B-specific and HCMV-specific expanded T-cell lines raised in parallel from the same donors (**Figure 2A**). The specific lysis of HHV-6B-pulsed target cells by HHV-6B-expanded T cells was lower than the specific lysis of HCMV-pulsed targets by HCMV-expanded T cells (*p* = 0.02, paired *t*-test, *n* = 2). These results are further evidence of differences between the HHV-6B and HCMV CD4 T cells.

To investigate phenotypic differences of the expanded HHV-6B- and HCMV-specific CD4 T cells, we assessed the CD4 memory subsets. We measured the frequency of various memory populations (using CD45RA and CCR7 markers) before and after the *in vitro* expansion, in total CD4^+^ and in the subset of CD4^+^ cells that were positive for HHV-6B or HCMV tetramers (**Figures 2B, C**). The same trend was observed in unstimulated PBMCs and in the *in vitro* expanded populations. HHV-6B-specific CD4 T cells have slightly higher amounts of cells in the central memory subset (CM, CCR7^+^CD45RA^−^; 1.2-fold in PBMCs and 2.7-fold in expanded cells), while HCMV-specific CD4 T cells have slightly increased amounts of the effector-memory subset (EM, CCR7^−^CD45RA^−^; 2.4-fold in PBMCs and 1.1-fold in expanded cells) (**Figure 2C**). These results are in line with phenotypic differences between the HHV-6B- and HCMV-specific CD4 T cells.

To investigate the functional differences between HHV-6B- and HCMV-specific CD4 T cells, we assessed the gene expression profiles of the cells by bulk RNA sequencing. Differential gene expression analysis was performed on HHV-6B-expanded and tetramer-sorted cells (three subjects), HCMV-expanded and tetramer-sorted cells (two subjects), and total unexpanded CD4^+^ cells from the same subjects as control (three subjects). Results are summarized in **Supplementary Table S2**. Principal component analysis (PCA) indicates that there are differences in the gene expression profiles of the three groups, with 63.4% of the differences explained by PC1 (expansion) and 13.9% by PC2 (antigen) (**Figure 2D**). The functional profile networks of tetramer-sorted HHV-6B and HCMV populations versus unexpanded total CD4^+^ T cells from matching donors showed indeed a strong component of responses to stimulation, reflecting the *in vitro* expansion (**Supplementary Figure S2**). Volcano plot comparing HHV-6B- and HCMV-specific cells shows 58 genes upregulated in HCMV and 144 genes upregulated in HHV-6B at fivefold difference and *p*_adj_ < 0.05 (**Figure 2E**; **Supplementary Figure S3**). The network for HHV-6B-expanded T cells showed an enhanced capacity for cell–cell and other extracellular interactions, including adhesion and interaction with soluble mediators when compared to the HCMV-expanded cells (**Figure 2F**), while the network for HCMV-expanded cells showed an enhanced cytotoxic gene expression profile (**Figure 2G**).

We also used single-cell sequencing to compare the gene expression data from tetramer-sorted HHV-6B-expanded T cells and HCMV-expanded T cells, and total unexpanded CD4 T cells (two subjects each). These data showed five major clusters: one cluster (FC2) corresponding to HCMV-expanded cells, two clusters (FC3 and FC5) corresponding to HHV-6B-expanded cells, one cluster corresponding to unexpanded CD4 T cells (FC1), and one cluster (FC4) containing cells that can be observed in all samples (**Figure 2H**). Analysis of the top differentially upregulated genes in each cluster (**Supplementary Table S3**) showed a marked cytotoxic signature along with chemotactic potential on the HCMV-expanded T cells (**Figure 2I**, FC2), while HHV-6B-expanded T cells were characterized by cytokine/chemokine recognition, other cell-surface receptors, and a mildly upregulated cytotoxic signature (**Figure 2I**, FC3). A subpopulation of HHV-6B-expanded cells (**Figure 2I**, FC5) had a functional signature of actively dividing cells, while cells in cluster FC4, observed in both populations, were characterized by strong mitochondrial metabolic activity (**Supplementary Figure S4A**). Among the genes more strongly expressed by HCMV-expanded cells are cytotoxicity mediators granzyme H, granzyme K, and granulysin; chemokines CCL3, CCL4, and CCL5; killer receptors KLRB1, KLRG1, and NKG7; and the transcription factor EOMES, the master regulator of the cytotoxic program (**Supplementary Figures S3**, **S4B**). All these indicate a more robust cytotoxic signature in the HCMV-expanded cells. In addition, these cells showed low or no expression of CD62L, CD27, and CCR7, which suggests that they belong to the effector-memory subset. In contrast, HHV-6B-expanded cells expressed higher levels of CD62L and CD27 than HCMV-expanded cells (**Supplementary Figures S3**, **S4B**), which suggests that these cells may resemble a central-memory subset. The HHV-6B-expanded T cells strongly express a different set of markers (**Supplementary Figures S3**, **S4B**), like TIMP1, LAG3, HOMER2, IL2RA, GRP15, LGALS3, ITGAX, and SELPLG, which may point to cell–cell interactions, migration, homing, and/or suppressor capabilities. These results suggest that HCMV-expanded cells have a strong cytotoxic profile characteristic of effector or terminal-effector cells, while HHV-6B-expanded cells have a less marked cytotoxic profile, along with other functional signatures.

### 3.3 TCR repertoires of HHV-6B- and HCMV-specific CD4 T cells

**Figure 3:**
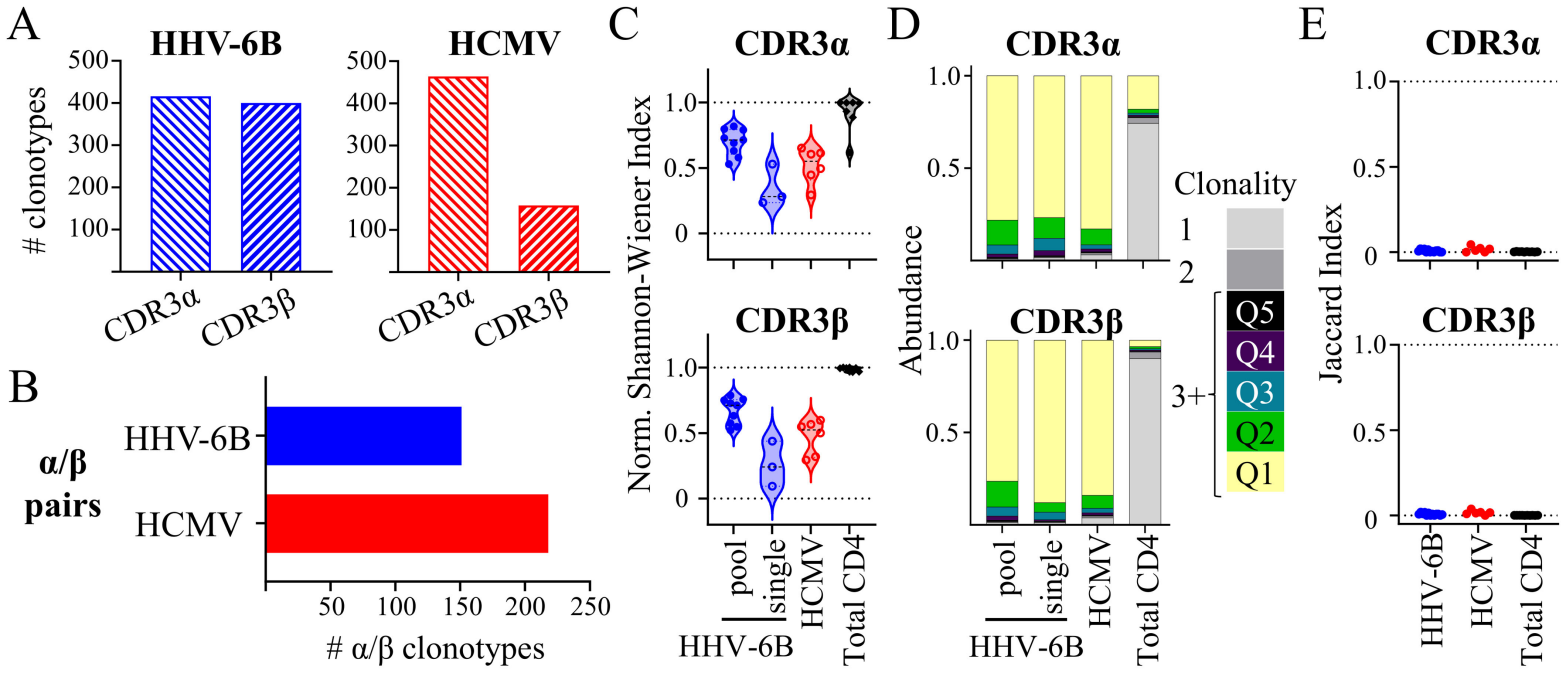
Diversity of HHV-6B-expanded, HCMV-expanded, and total unexpanded CD4 TCR repertoires. **(A)** Total number of CDR3α and CDR3β clonotypes identified by bulk TCR sequencing. **(B)** Total number of TCRα/β pairs identified by single-cell sequencing. **(C)** Repertoire diversity: Normalized Shannon–Wiener index for each subject in each antigen group: HHV-6B (blue circles), HCMV (red circles), and unexpanded total CD4 (black diamonds). **(D)** Repertoire clonality: The average abundance (as a fraction of the total) of clonotypes present as singletons (1), doubletons (2), or higher order (3+) for each subject in the same antigen groups described in C; for higher order, the abundance of clonotypes was presented by quantiles: Q1 (top 20%), Q2 (21%–40%), Q3 (41%–60%), Q4 (61%–80%), and Q5 (81%–100%). **(E)** Repertoire overlap: Jaccard index was calculated for each pair of subjects for each antigen group (HHV-6B pool, HCMV, and unexpanded total CD4).

We next investigated the TCR repertoire of the tetramer-sorted HHV-6B- and HCMV-specific CD4 T cells expanded *in vitro* with the epitopes listed in **Table 1**. Tetramer-sorted T cells from five subjects for HHV-6B and three subjects for HCMV were used for bulk TCR sequencing, and tetramer-sorted T cells from three subjects each for HHV-6B and HCMV were used for single-cell TCR sequencing. Overall, a total of 416 CDR3α and 400 CDR3β in HHV-6B-specific samples, and 232 CDR3α and 79 CDR3β in HCMV-specific samples were identified by bulk sequencing (**Figure 3A**). The single-cell data identified a total of 149 HHV-6B-specific and 215 HCMV-specific α/β pairs (**Figure 3B**). The data are summarized in **Supplementary Tables S4A, B**, and all clonotypes are shown in **Supplementary Tables S4C, D**.

The diversity of the repertoire was assessed using the normalized Shannon–Wiener index plots (**Figure 3C**). A reduced diversity of all *in vitro* expanded HHV-6B- and HCMV-specific CD4 T cells compared to the total unexpanded CD4 T cells was observed. The pool of HHV-6B showed higher diversity than HHV-6B single peptides, consistent with the presence of subpopulations of T cells recognizing the different epitopes present in the pool. Some clonotypes were strongly expanded by the antigen stimulation (**Figure 3D**). The unexpanded CD4 TCR repertoires were mostly constituted by clonotypes that are present as singletons (clonality of 1, **Figure 3D**). Expanded clonotypes (clonality of 3+) dominated the repertoire of *in vitro* expanded samples, with a strong enrichment of the top 20% of the clonotypes (**Figure 3D**, Q1), confirming that the *in vitro* expansion results in the enrichment of a few antigen-specific clonotypes. We also assessed the TCR repertoire overlap within each group (HHV-6B, HCMV, or unexpanded CD4) using the Jaccard index calculated for pairs of subjects (**Figure 3E**); results showed a small overlap between the TCR repertoires of different subjects within a group, which suggests that the repertoires are mainly private.

### 3.4 Public HHV-6B and HCMV TCRs

**Figure 4:**
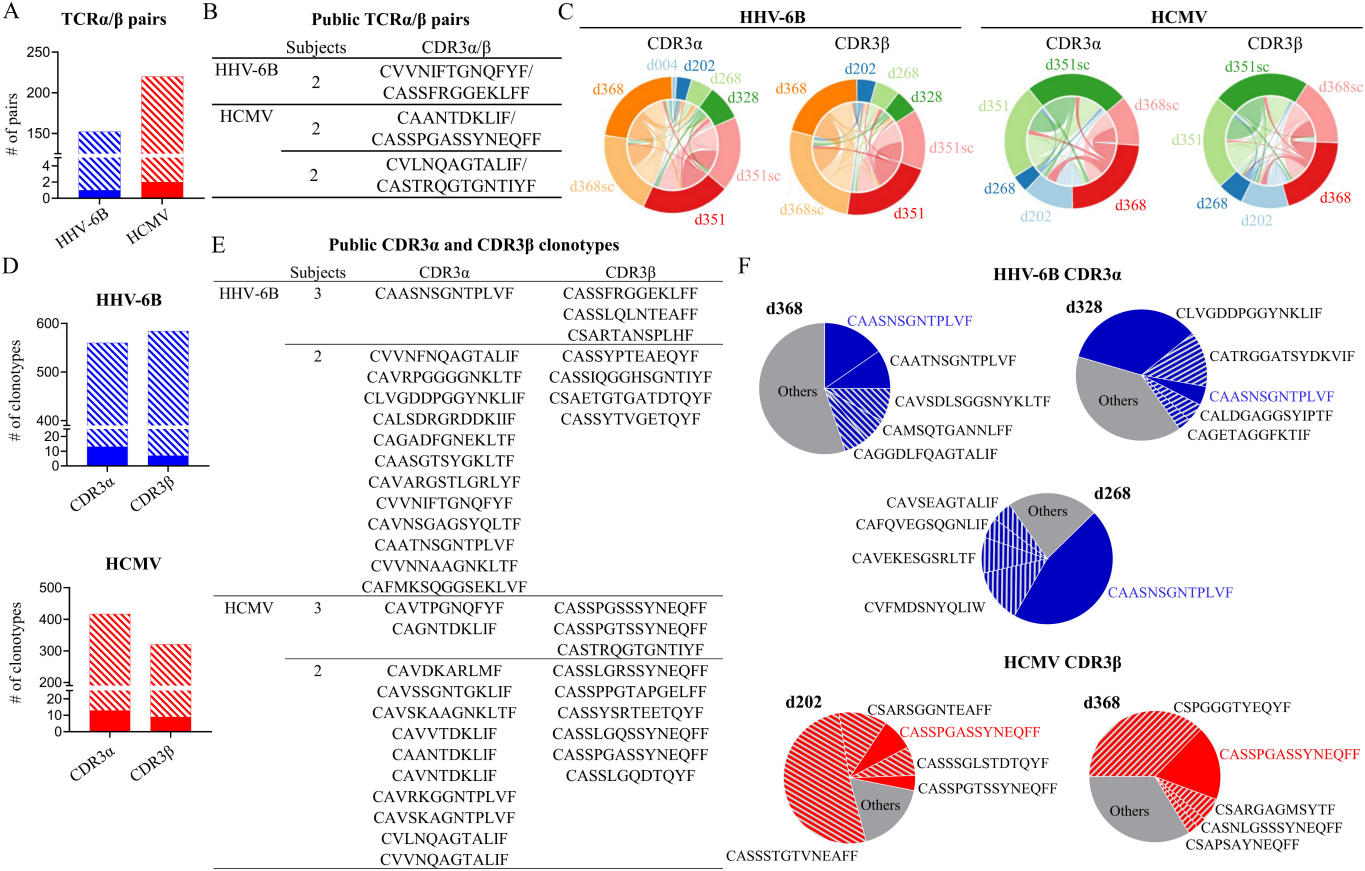
Publicity of the HHV-6B and HCMV repertoires. **(A)** Total TCRα/β pairs identified, showing the fraction corresponding to public α/β clonotypes as a solid fill (0.5% for HHV-6B and 0.9% for HCMV). **(B)** Public TCRα/β pairs identified and number of subjects sharing each pair. **(C)** Summary of shared CDR3 clonotypes among different subjects (pairwise overlap Circos plot; “sc” next to subject ID denotes single-cell sequencing; all others are bulk sequencing). **(D)** Total unique CDR3α (547 for HHV-6B and for 403 for HCMV) or CDR3β (576 for HHV-6B and 314 for HCMV) identified in the combined data (uncoupled single-cell data and bulk data from all subjects; data from the same subject were combined to obtain unique sequences per subject). The public clonotypes (observed in two to three subjects) are shown as a solid fill (for HHV-6B: 2.3% CDR3α and 1.2% CDR3β; for HCMV: 3.1% CDR3α and 2.8% CDR3β). **(E)** Public CDR3α and CDR3β clonotypes and number of subjects sharing these sequences. **(F)** Examples of abundant public CDR3s: top five clonotypes from selected subjects (colored pie slices) showing public clonotypes (solid filled slices). Blue for HHV-6B CDR3α sequences and red for HCMV CDR3β sequences; public abundant clonotype sequences highlighted in color.

When different subjects share the same CDR3 amino acid sequence, it is referred to as public. Public TCRs (individual TCRα or TCRβ, or α/β pairs) have been widely described, and they have drawn interest because of their utility in following T-cell populations across subjects and because they may be associated with the manifestation and outcome of a disease (8). We looked for identical CDR3s shared across our study subjects (**Figure 4**). In our single-cell data, we identified three public TCRα/β pairs, one specific for HHV-6B and two for HCMV, representing up to 1% of all the unique pairs for each virus in all donors (**Figures 4A, B**). The public TCRα/β pairs represent 1.2%–1.9% of the unique pairs in each of the subjects in which they were found. Likewise, public individual TCRα or TCRβ chains represent 1%–6% of all unique sequences per subject. Moreover, after combining all the public TCRα or TCRβ within a subject, the percentage was up to 45% of the total unique clonotypes of a subject (**Supplementary Table S4**). The *in vivo* frequency of the public clonotypes was not assessed because of the overall low frequency of the tetramer-specific cells in *ex vivo* unstimulated samples. Comparing the individual CDR3α and CDR3β (Circos plots in **Figure 4C**), shared clonotypes among two or three different subjects can be observed as thin lines connecting different subjects, while a larger overlap was observed between samples from the same subject (thick lines connecting single-cell and bulk samples from the same subject). Since the single-cell and bulk samples were obtained 8–14 months apart, a high degree of overlap indicates the presence of clonotypes that have persisted in time for a given subject (**Supplementary Figure S5A**). For further analysis, we combined both bulk and single cell data from the same subjects and listed the unique clonotypes. Analyzing all unique clonotypes of all subjects, we identified 41 CDR3α and CDR3β public clonotypes from both HHV-6B and HCMV, which represent up to 4% of the CDR3α or CDR3β sequences (**Figure 4D**).

All public CDR3α and CDR3β clonotypes are summarized in **Figure 4E**. Some public CDR3 clonotypes were among the most abundant in multiple subjects (colored blue and red in **Figure 4F**). For example, HHV-6B public CDR3α CAASNSGNTPLVF was among the top five most abundant clonotypes in d268, d328, and d368 (**Figure 4F**, blue pies), representing 47%, 7.5%, and 18.8% of the total counts, respectively. Likewise, HCMV public CDR3β CASSPGASSYNEQFF was also among the most abundant in d202 and d368 (**Figure 4E**, red pies), representing 8% and 18% of the total counts, respectively. Besides these highly abundant public clonotypes, low-abundance public clonotypes were also observed. For example, the public HCMV CDR3α CAGNTDKLIF was observed in three subjects d202, d351, and d368 at abundances of 0.09%, 0.04%, and 0.37% of the total counts. Likewise, the public HHV-6B CDR3β CSARTANSPLHF was observed in d368 at 4.3% of the total counts, but in d268 and d328, it was observed at 0.03% and 0.8% of the total counts, and HCMV CDR3β CASTRQGTGNTIYF was observed in d202, d268, and d368 at 0.81%, 0.09%, and 0.16% of the total counts, respectively. Public clonotypes were among those observed in the same subject at different times. For subject d368, the public HHV-6B CDR3α CAASNSGNTPLVF, CAATNSGNTPLVF, and CVVNIFTGNQFYF and CDR3β CASSFRGGEKLFF as well as the public HCMV CDR3α CAVRKGGNTPLVF were all observed in three samples collected within November 2018 and January 2020 (**Supplementary Figure S5B**). The public HHV-6B clonotypes, however, were not found in unexpanded total CD4 or PBMC samples from the same subjects, suggesting that these clonotypes were present at low frequencies and that our data were not deep enough to observe them without expansion. This observation is consistent with very low frequencies of HHV-6B-specific T-cell responses measured directly *ex vivo* [for example, 14 responding cells per 10^6^ PBMCs as reported in reference (48)]. For HCMV, we found several public clonotypes (CASSPGASSYNEQFF, CASSLGQSSYNEQFF, CAVNTDKLIF, and CVVNQAGTALIF) that were present in both expanded T cells and unexpanded total CD4 T cells from the same subjects, as well as private HCMV clonotypes CAVKKAGNTPLVF and CSPGGGTYEQYF, consistent with the large proportion of HCMV-specific T cells in subjects (28).

One mechanism for generating public TCR is sequence convergence, i.e., the same CDR3 amino acid sequence is encoded by different nucleotide sequences within a subject or among different subjects (8, 10, 11, 88). We observed that 8% of the HHV-6B and 10% of the HCMV CDR3s amino acid sequences in the dataset were encoded by at least two and up to five different nucleotide sequences (**Supplementary Figure S6A**). Among sequences that show convergence, 22% of HHV-6B and 29% of HCMV were observed in different subjects (i.e., public); these include all the sequences reported in **Figure 4E**. Convergent sequences showed a wide abundance range within and among subjects (**Supplementary Figure S6B**). In **Supplementary Figure S6C**, we show a few examples of convergence in our public clonotypes. CAASNSGNTPLVF was encoded by five different nucleotide sequences in three different subjects, CSARTANSPLHF was encoded by four nucleotide sequences in three subjects, and CASSFRGGEKLFF and CASSPGASSYNEQFF were encoded by three nucleotide sequences in three subjects each (**Supplementary Figure S6C**). All these observations are compatible with the notion of the generation of publicity by sequence convergence.

Finally, the HHV-6B TCR dataset includes clonotypes with specificity for any of the six different epitopes present in the peptide and tetramer pools used for expansion and sorting. To identify the epitope associated with specific HHV-6B clonotypes, single-tetramer-sorted cells from one subject (d368) were analyzed by bulk sequencing. Sufficient cells could be collected only for three tetramers: U11, U56, and U85. In these three samples, we identified 47 CDR3α and 54 CDR3β clonotypes (**Supplementary Table S4**). Among these were some of the public clonotypes defined before, including the CDR3α and CDR3β that conform to the HHV-6B-specific α/β pair (CVVNIFTGNQFYF and CASSFRGGEKLFF, respectively), which were present in the U11-sorted sample, and CDR3α CAASNSGNTPLVF and CDR3β CSARTANSPLHF, which were identified in the U85-sorted sample. No public sequences were identified in the U56-sorted sample.

Overall, the HHV-6B and HCMV TCR repertoire data showed the presence of public clonotypes (individual TCRα, individual TCRβ, and α/β pairs). In some cases, these public clonotypes were among the most abundant in the *in vitro* expanded T cells, and for a few HCMV clonotypes, they were also observed in unexpanded total CD4 T cells.

### 3.5 Public TCRs in large datasets of unsorted peripheral blood T-cell repertoires

**Figure 5:**
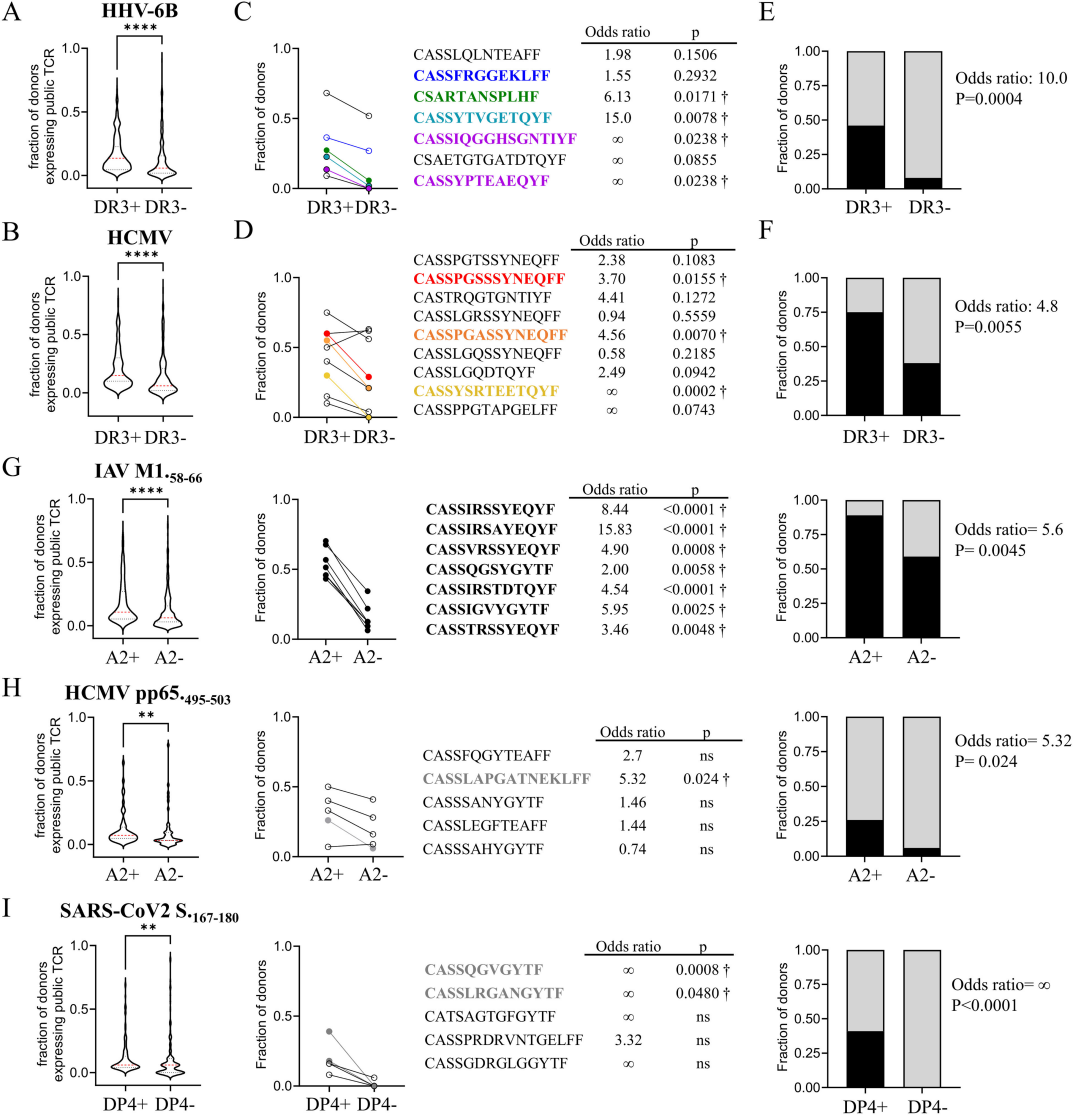
Publicity of HHV-6B and HCMV CDR3β clonotypes in datasets of unsorted TCRs. We looked for our public HHV-6B and HCMV CDR3β clonotypes in reported datasets that were not antigen-specific (methods), and selected CDR3β clonotypes that were observed in two or more subjects; subjects were grouped according to DRB1*03:01presence (DR3+) or absence (DR3−). **(A)** Fraction of subjects in which a given HHV-6B-associated CDR3β clonotype was observed in the DR3+ or DR3− groups; statistical analysis by paired *t*-test. **(B)** Same as **(A)** but for HCMV-specific clonotypes. **(C)** Fraction of subjects in the DR3+ or DR3− groups expressing the HHV-6B CDR3β public clonotypes described in this work (observed in at least two of our study subjects). The top public clonotypes are shown as well as odds ratio and *p*-value (Fisher’s exact test) for each one; significant ones are marked by a dagger. **(D)** Same as **(C)** but for HCMV CDR3β public clonotypes. **(E)** Fraction of subjects expressing at least one of the significant CDR3β HHV-6B clonotypes in **(C)** (black) or not expressing any (gray); combined odds ratio and *p*-value (Fisher’s exact test) are shown. **(F)** Same as **(E)** but for HCMV. **(G–I)** The same analysis was done for previously published repertoires recognizing the following: **(G)** AIV M1.58–66 presented by A*02:01 (77); **(H)** HCMV pp65.495-503 (78); and **(I)** SARS-CoV2 S.167-180 (79). Violin plots' statistical analysis by paired t-test (**p < 0.005; ****p < 0.0001).

To extend our assessment of the publicity of our HHV-6B and HCMV clonotypes, we compared our data to publicly available TCR CDR3β datasets. We used a dataset of total PBMC or total CD4 T cells from three DRB1*03:01 and two non-DRB1*03:01 subjects reported by Soto et al. (74), and a dataset from total PBMCs from 13 DRB1*03:01 and 50 non-DRB1*03:01 subjects reported by Nolan et al. (75). In total, these two datasets comprise almost 15,000,000 CDR3β clonotypes. Of the HHV-6B and HCMV CDR3β clonotypes described above, 40% of the HHV-6B-specific sequences (230 out of 576) and 57% of the HCMV-specific sequences (179 out of 314) were public, i.e., present in our dataset and in at least one of the published datasets (summarized in **Supplementary Table S5**). For both HHV-6B and HCMV, the fraction of subjects expressing public CDR3β was significantly higher in the DRB1*03:01-positive subjects as compared to DRB1*03:01-negative subjects (**Figures 5A, B**). We looked specifically at the CDR3β clonotypes that we defined as public based on our data, and confirmed that, in most cases, they were more frequently observed in the DRB1*03:01-positive population (**Figures 5C, D**). For example, the HHV-6B-associated CDR3β clonotypes CSARTANSPLHF and CASSFRGGEKLFF were 6.1 and 1.6 times more likely to be observed in DRB1*03:01-positive samples (*p* = 0.017 and *p* = 0.29, respectively), and the HCMV-associated CDR3β clonotype CASSPGASSYNEQFF was 4.6 times more likely to be observed in DRB1*03:01-positive samples (*p* = 0.007). The fraction of subjects expressing these individual clonotypes was 0.27, 0.36, and 0.55, respectively. Combining multiple public clonotypes that individually were already significantly enriched in the DRB1*03:01 population (marked by a dagger in **Figures 5C, D**), we were able to increase the coverage of the DRB1*03:01 population that was expressing at least one of these clonotypes (the fraction of subjects were now 0.55 and 0.75 for HHV-6B and HCMV, respectively; **Figures 5E, F**), while maintaining a discriminatory power between the positive and negative populations (odds ratios of 14.4 for HHV-6B and 3.4 for HCMV). To assess how this publicity compares to other known viral epitopes, we analyzed previously reported public TCR clonotypes. We examined the CDR3β repertoire recognizing the well-known influenza epitope M1.58–66 bound to A*02:01 published by Song et al. (77). The public clonotypes were more frequently observed in the A*02:01-positive population (**Figure 5G**). The top clonotypes combined were found in almost 90% of the subjects in the A*02:01-positive group. A known epitope from HCMV pp65.495–503 bound to A*02:01 (78) was also analyzed. The public CDR3β associated with this epitope was also observed more frequently in the A*02:01-positive population (**Figure 5H**), although only one of the clonotypes was statistically significant, but with only 25% coverage of that population. For the MHC-II epitope SARS-CoV2 S.167–180 bound to DPA1*01:03/DPB1*04:01 (DP4) reported by Mudd et al. (79), the significant clonotypes combined were observed in 40% of the DP4-positive population and in none of the DP4-negative subjects (**Figure 5I**). For the cross-reactive clonotypes recognizing SARS-CoV2 S.815–829 peptide bound to DP4 (65), we did not observe any difference in frequency in the DP4-positive vs. DP4-negative populations (**Supplementary Figure S7**). Overall, the public clonotypes recognizing different viral epitopes were more frequently observed in subjects with a partial HLA match, although the sets showed variable population coverage and HLA specificity.

To verify the antigen specificity and HLA restriction of the clonotypes, we searched for our HCMV and HHV-6B clonotypes within datasets of TCRs with a known antigen specificity. In a dataset of CDR3β clonotypes from unsorted T cells *in vitro* expanded with peptide pools from common-cold coronaviruses’ spike protein from two DRB1*03:01-positive and five DRB1*03:01-negative subjects (65), we only found two HHV-6B (CASSFGQNTEAFF and CASSHRGGETQYF) and one HCMV (CASSPYNEQFF) identical clonotype (all in DRB1*03:01-negative subjects). None of the HHV-6B CDR3β clonotypes were present in a list of 1,434 HLA-restricted and antigen-specific CDR3β downloaded from VDJdb (76) for the HLA alleles DRB1*01, 03, 04, 07, and 15, and DRB3*02 and 03. One of our HCMV clonotypes was reported in that dataset (CASSPYNEQFF), for the peptide pp65 LLQTGIHVRVSQPSL presented by DRB1*15:01. None of our HCMV CDR3β clonotypes were found in a list of 164 HCMV-associated CDR3β clonotypes reported by Emerson et al. (14), which comprises 45 clonotypes assigned to MHC-I and 119 clonotypes with unknown HLA, or in a list of 926 previously reported CDR3β clonotypes recognizing HCMV antigens also used by Emerson et al. in their analysis (the restriction of these TCRs was mostly MHC-I, ~90% of the sequences). Likewise, none of our clonotypes were found in the pp65-specific CD4^+^ T-cell repertoire reported by Lyu et al. (60).

These results show that the HHV-6B- and HCMV-associated public CDR3β clonotypes identified here can be found in unexpanded and unselected repertoires from subjects expressing mainly DRB1*03:01 and were mostly absent from other antigen-specific repertoires regardless of the HLA.

### 3.6 Related CDR3 amino acids sequences: convergence groups and CDR3 motifs

**Figure 6:**
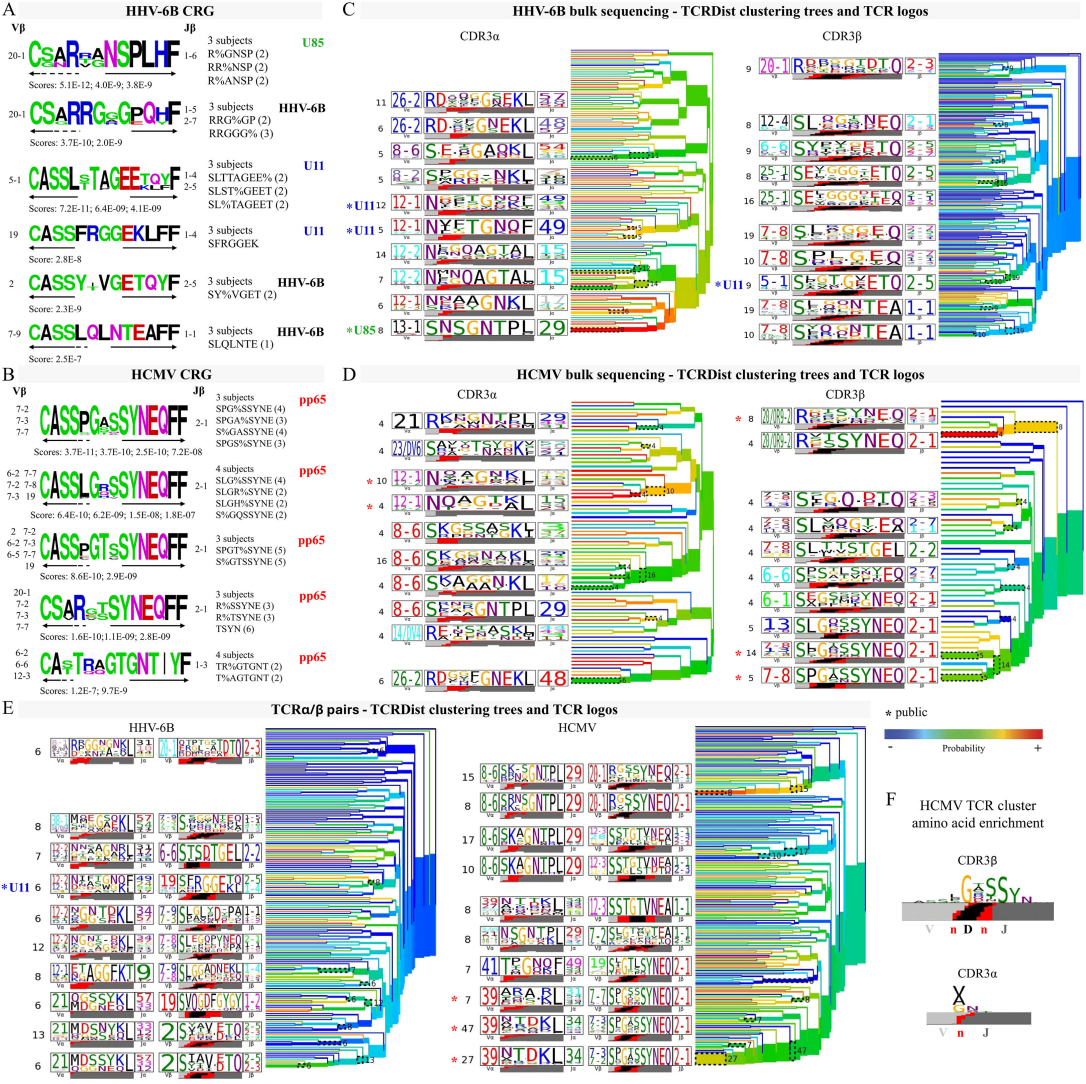
Groups of related TCRs identified using clustering algorithms. **(A, B)** Sequence logos of CDR3β convergent recognition groups identified using the GLIPH2 algorithm in HHV-6B **(A)** and HCMV **(B)** data. Each logo was obtained using the sequences in the convergence groups listed to the right, with the number of different sequences in parenthesis. The number of different subjects in each group is indicated above the patterns or motifs. The Vβ and Jβ genes in those sequences are on each side of the logo, and the arrows indicate the residues that come from each gene. The scores for each pattern or motif are indicated below the logo. For HHV-6B, when sequences from individual tetramer-sorted samples were part of a group, the antigen specificity is indicated (U11 in blue, U85 in green); otherwise, it is shown as HHV-6B. **(C)** Average-linkage dendrograms of TCRDist clusters and logos of CDR3α and β for HHV-6B (five subjects, bulk sequencing). **(D)** Same as **(C)** but for HCMV (three subjects each bulk sequencing). **(E)** Average-linkage dendrograms of TCRDist clusters and logos of α/β pairs from the single-cell sequencing data for HHV-6B or HCMV (three subjects each). In **(C–E)**, * indicates clusters containing public sequences; for HHV-6B, the specific epitope is indicated if known. **(F)** CDR3 amino acid enrichment in CDR3α and β motifs for a representative HCMV TCR cluster.

TCR clonotypes that share sequence patterns or motifs involved in recognizing the pMHCs can be expected to recognize the same epitope (73, 77, 78, 89). Therefore, we sought to identify subsets of related clonotypes that may be associated with the same antigen specificity within the overall TCR repertoires of HHV-6B- and HCMV-specific T cells. We used the GLIPH2 (89) and TCRDist (73) algorithms to find clusters of similar or related clonotypes. GLIPH2 clusters CDR3β clonotypes in convergent recognition groups (CRGs) defined by conserved patterns and motifs identified in the related sequences (**Supplementary Table S6**). Using GLIPH2, 101 antigen-specific CRGs were obtained (at a V-gene bias *p* < 0.05), representing 67 patterns and motifs (39 associated with HHV-6B and 28 associated with HCMV). Of the 37 HHV-6B CRGs, 19 were observed in more than two subjects, and the number of unique sequences per group was 2 to 3 (**Supplementary Table S6A**). Of the 28 CRGs associated with HCMV, 23 were observed in more than two subjects, and these groups contained at least two and up to six unique sequences (**Supplementary Table S6B**). Representative CRGs containing sequences observed in two or more subjects are shown as sequence logos in **Figure 6A** for HHV-6B and **Figure 6B** for HCMV. For three of the HHV-6B groups, we assigned the antigen specificity-based TCRβ clonotypes observed in the single-tetramer-sorted samples (indicated in green for U85 or blue for U11). TCRDist clusters clonotypes based on a similarity-weighted mismatch distance-based metric and generates motifs for both CDR3α and CDR3β (73). **Figure 6C** shows the average-linked dendrograms of the clusters obtained using the TCRDist algorithm, showing gene usage and CDR3 logos obtained for the analysis HHV-6B data from bulk sequencing (five subjects) and single-cell sequencing (three subjects). **Figure 6D** shows the corresponding data for HCMV (three subjects each).

Using these tools, we found closely related clonotypes that may have the same specificity shared by multiple subjects. For example, for HHV-6B, the public CDR3β CSARTANSPLHF (**Figure 4E**) was part of the CRG with the pattern R%ANSP, along with CSARRANSPLHF. This clonotype, in turn, was also in CRGs with the patterns RR%NSP and R%GNSP (**Figure 6A**). One of the clonotypes of CRG R%GNSP was CGNRVGNSPLHF, the most abundant clonotype identified in the U85 single-tetramer-sorted sample. In addition, CGNRVGNSPLHF pairs with the highly abundant public α chain CAASNSGNTPLVF (described in **Figure 4F**). TCRDist identified the highly significant motif SNSGNTPL (**Figure 6C**), which corresponds to this CDR3α. CGNRVGNSPLHF can also pair with CAATNSGNTPLVF, which has only one substitution at position 4 compared to CAASNSGNTPLVF. This CDR3α also pairs with CSARRGGGPQHF (GLIPH2 patterns RRG%GP and RRGGG%). Taking all these observations together, we believe that all these CDR3 clonotypes are involved in recognizing the U85 peptide.

For the public HHV-6B TCRα/β pair probably recognizing the U11 peptide (**Figure 4B**), TCRDist identified clusters of related sequences for both the α and β chains (**Figure 6C**). A second different cluster of clonotypes associated with U11 was identified. These clonotypes were in three CRGs (patterns SLTTAGEE%, SL%TAGEET, and SLST%GEET, **Figure 6A**) containing similar CDR3β shared by three subjects. One of the clonotypes (CASSLTTAGEEKLFF) was identified in the U11 single-tetramer-sorted sample. In single-cell data, this sequence pairs with CASSGAGSYQLTF. Other clonotypes in these CRGs pair with similar CDR3α: CASSLSTAGEETQYF with CAHSGAGSYQLTF (d368) and CASSLSTPGEETQYF with CAPSGAGSYQLTF (d351) and may share the same epitope specificity.

For one of the HCMV public TCRα/β pairs (**Figure 4B**), the CDR3β CASSPGASSYNEQFF appeared to be related to other public CDR3β clonotype reported in **Figure 4E**. When we used the clustering algorithms, we found at least 10 CRGs using GLIPH2 (**Figure 6B**) as well as at least four significant motifs identified by TCRDist (**Figure 6D**). All these CRGs and motifs have a strong bias to Jβ2–1 usage, and there is a strong preference for G in position 6 and S in position 8 of these CDR3β as shown in the amino acid enrichment analysis (**Figure 6E**). The CDR3α in this public TCRα/β pair CAANTDKLIF (**Figure 4B**) was also part of at least two motifs identified by TCRDist in the single-cell data (**Figure 6D**, right panel). The amino acid enrichment analysis shows a preference for V/A/G on position 3 (**Figure 6F**); some of the public CDR3α reported in **Figure 4E** fit this motif.

### 3.7 Validation of selected TCRα/β pairs

We studied selected TCR clonotypes recognizing HHV-6B and HCMV epitopes (**Table 2**) to confirm the α/β pairing and the specificity and affinity of epitope recognition, and to get insights into the TCR–pMHC interaction. We selected representative TCRα/β pairs containing shared identical and/or highly similar CDR3α and/or CDR3β for which a known pairing from single-cell data was available. For HHV-6B, the selected clonotypes include TCRs associated with U11 and U85. Each TCR α and β chain was cloned into lentivirus vectors and transduced into Jurkat T cells [ (78), see methods for details].

**Table 2:** Selected TCRα/β recognizing HHV-6B or HCMV epitopes.

Virus	TCR Id	CDR3^1^	Sequence^2^	TRV^3^	TRJ^3^	Public^4^	GLIPH2^5^	TCRDist^6^	Validated^7^
HHV-6B	U11.1	α	CVVNIFTGNQFYF	12-1	49	α, β^8^, α/β	1(3)	5(α), 6(α/β)	+
		β	CASSFRGGEKLFF	19	1-4				
	U11.2	α	CASSGAGSYQLTF	36-DV7	28		6(3)	0	+
		β	CASSLTTAGEEKLFF	5-1	1-4				
	U85.0	β	CSARTANSPLHF	20-1	1-6	β^8^	4(3)	0	
	U85.1	α	CAASNSGNTPLVF	13-1	29	α	4(3)	8(α)	+
		β	CGNRVGNSPLHF	20-1	1-6				
	U85.2	α	CAATNSGNTPLVF	13-1	29	α	4(3)	8(α)	+
		β	CSARRGGGPQHF	20-1	1-5				
HCMV	pp65.0	α	CGAANTDKLIF	34	34		0	0	+
		β	CSARGAGMSYTF	20-1	1-2				
	pp65.1	α	CVLNQAGTALIF	12-1	15	α, β^8^, α/β	3(4)	4(α)	
		β	CASTRQGTGNTIYF	6-2	1-3				
	pp65.2	α	CAVKKAGNTPLVF	8-6	29		0	0	
		β	CSPGGGTYEQYF	20-1	2-7				
	pp65.3	α	CAANTDKLIF	39	34	α, β^8^, α/β	14(3)	14(β), 27(α/β)	+
		β	CASSPGASSYNEQFF	7-3	2-1				

^1^ CDR3 subunit. ^2^ Clonotype amino acid sequence; underlined are insertions. ^3^ V and J genes. ^4^ Identical CDR3β, CDR3β, or CDR3α/β pair found in subjects in this study. ^5^ Number of related CDR3β found using clustering algorithm GLIPH (with number of subjects shown in parenthesis). ^6^ Number of related CDR3α, CDR3β, or CDR3α/β sequences found using TCRDist (with number of sequences per cluster shown in parenthesis). ^7^ Clonotype validated experimentally in this work. ^8^ Identical CDR3β also found in previously published studies.

**Figure 7:**
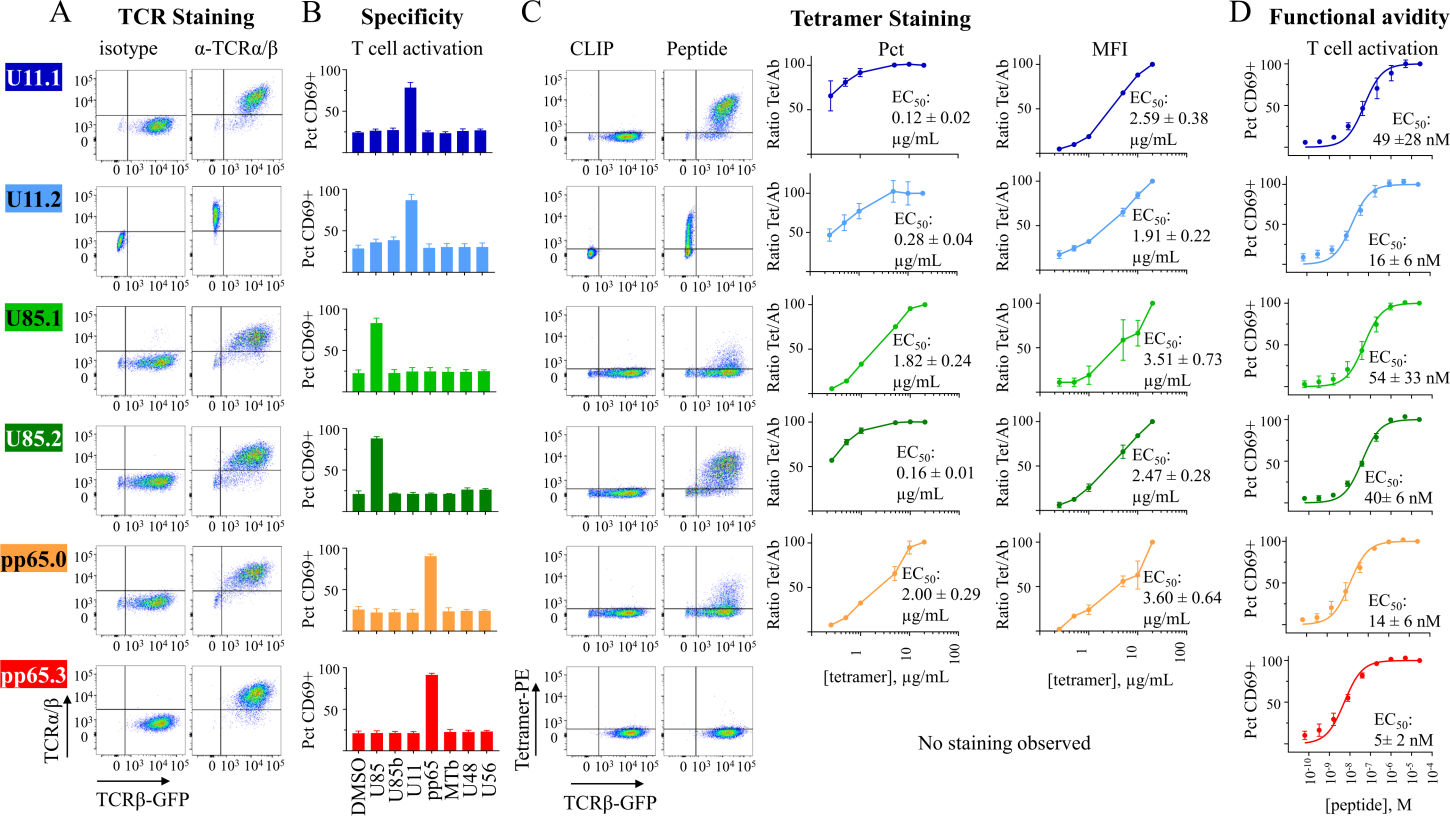
Validation of TCR expression, pairing of α and β subunits, and specificity of single-TCR transduced cell lines. **(A)** TCRα/β expression on surface, measured by flow cytometry using IP26 antibody (that recognizes the heterodimer TCRα/β) and GFP (which is expressed by TCRβ, except for U11.2, which was produced as a P2A construct in a pCDH-puromycin vector). **(B)** Response of transduced cell lines (measured as CD69 surface expression) to different DRB1*03:01 peptides (2 μg/mL) presented by the DRB1*03:01/DRB3*01:01-expressing cell line Vavy. **(C)** Tetramer staining of transduced cell lines with their specific DRB1*03:01 tetramer or the control CLIP-DRB1*03:01 tetramer; representative dot plots at a tetramer concentration of 5 µg/mL; dose–response curves of percentage of positive cells and MFI (0.25–20 µg/mL). **(D)** Functional avidity (EC_50_, nM) of TCRs measured as CD69 expression at different doses of the specific peptide (0.13 ng/mL to 50 μg/mL). Flow cytometry gating strategy is shown in **Supplementary Figure S8A**.

Jurkat T cells transduced with paired TCRα and TCRβ subunit genes in all cases expressed the assembled TCRα/β complex on the cell surface (**Figure 7A**) as detected using the monoclonal antibody (IP26) recognizing the α/β heterodimer; no staining was observed in non-transduced cells (**Supplementary Figure S8B**). We sorted cells expressing the TCRα/β and used them for functional assays. In these assays, we measured the expression of CD69 on surface after peptide stimulation using the DRB1*03:01-expressing cell line Vavy as presenting cells. All transduced TCRs were activated by their specific peptide and not by any other of the DRB1*03:01 peptides tested (**Figure 7B**). Transduced cells recognized the corresponding DRB1*03:01 tetramer and not the CLIP-DRB1*03:01 tetramer, except for pp65.3 (**Figure 7C**). The recognition was variable at the tested tetramer concentration of 5 µg/mL, showing that there are differences in the tetramer avidity for the different TCRs. Dose–response for the tetramer binding experiment using 0.1 µg/mL up to 20 µg/mL (**Figure 7C**) showed high avidities for U11.1, U85.2, and U11.2 with saturation at both percentages of positive cells and MFI levels, and lower avidities for U85.1 and pp65.0. The pp65.3 tetramer showed no staining at any dose tested. Dose–response using the CD69 expression assays (**Figure 7D**) showed functional avidities within the ~5–50 nM range, which suggests intermediate to high affinity in the recognition of the pMHC.

**Figure 8:**
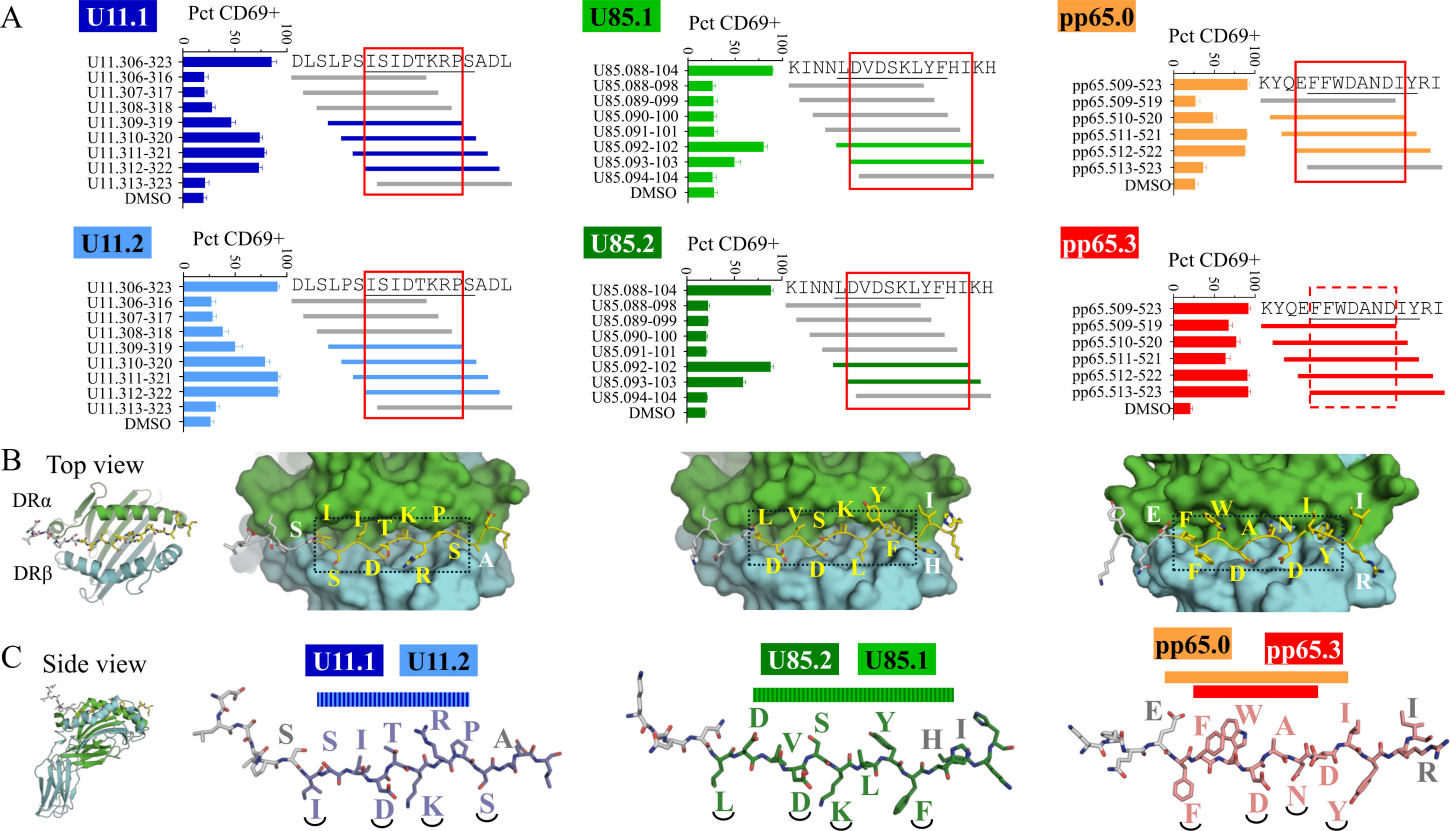
Insights into pMHC–TCR interaction. **(A)** Mapping of the minimal peptide necessary for TCR recognition of their pMHC using a CD69 expression assay (bar graphs). Full-length peptide (top) and a series of 11mer truncated peptides that scanned the whole sequence were used. The predicted binding core for DRB1*03:01 is underlined in the full-length peptide. Truncated peptides that were positive are in color and a box indicates the residues necessary for TCR recognition. **(B, C)** AlphaFold2 models of DRA/DRB1*03:01 complexes with U11, U85, and pp65 peptides. **(B)** Top view: MHC surface showing the peptide core residues in yellow, and flanking residues in white. The box encloses the predicted binding core. **(C)** Side view of the peptides (core in color, flanking residues in gray); residues at p1, p4, p6, and p9 (underlined) are bound in the pockets in the MHC binding groove; other residues may be available for TCR interaction.

We used truncated peptides covering each epitope peptide to gain insight into the way the TCRs recognize the pMHCs (**Figure 8A**). Using the CD69 expression assay, we determined the minimal peptide necessary for TCR recognition. Both U11.1 and U11.2 responded to peptides containing the sequence ISIDTKRP, which are p1–p8 in the predicted binding core. Both U85.1 and U85.2 responded to peptides containing the sequence DVDSKLYFHI, which are residues p2–p9 of the predicted binding core, along with p10–p11. pp65.0 responded to peptides containing the sequence EFFWDANDI, which are residues p(−1)–p8 of the predicted binding core. pp65.3 responded to peptides containing FFWDAND, residues p1–p7 of the predicted binding core. To illustrate likely MHC and TCR contacts, we used AlphaFold2 to produce models of pMHCs (**Figure 8B**). We selected top models for HHV-6B and HCMV displaying the peptides in the predicted binding registers and preserving the canonical hydrogen bonding. Residues at p1, p4, p6, and p9 (underlined in **Figure 8C**) are bound in pockets in the MHC binding groove; other residues are considered available for TCR interaction. The model for U11 shows that residues p2, p3, p5, p7, and p8 are all pointing up, which agrees with the reactivity observed. The model for U85 shows that besides p2, p3, p5, and p8, residues at p10 (H) and p11 (I) are also available for TCR interaction, which is consistent with the reactivity observed. The model for pp65 shows that besides p2, p3, p5, p7, and p8, residue at p−1 (E) is available for TCR interaction, which is consistent with the reactivity observed for pp65.0.

Lastly, to explore the potential cross-reactivity of the U11-, U85-, and pp65-associated public TCRs, we performed a comparison of these epitopes with their homologs in the related roseoloviruses HHV-6A and HHV-7 (**Supplementary Figure S9**). Sequence alignment of the HHV-6B proteins and their positional homologs in HHV-6A and HHV-7, along with information from binding predictions and predicted T cell contacts, showed that the HHV-6B peptide U11.306–323 has enough homology with its HHV-6A homolog to retain binding to MHC and preserve T-cell contacts, as indicated by the predicted MHC binding and predicted TCR interaction scores (**Supplementary Table S7**). The same is not true for the HHV-7 and HCMV homologs, which are not predicted to bind and there are no preserved T-cell contacts (**Supplementary Table S7**). For HHV-6B U85.88-104, the HHV-6A homolog is predicted to bind and retain the TCR contacts but not the HHV-7 homolog (**Supplementary Table S7**). For HCMV pp65.509-523, there is very low homology with the protein from the three roseolovirus homologs. All this indicates that cross-reactivity between U11 and U85 TCR with HHV-6A homologs is possible and the HCMV TCR seems to be very specific.

## 4 Discussion

We studied the TCR repertoires and functional profiles of CD4 T cells and cytotoxic capacity that recognize epitopes derived from two herpesviruses, HHV-6B and HCMV. T-cell populations from peripheral blood of subjects that expanded *in vitro* in response to six HHV-6B peptides or one HCMV peptide were isolated by MHC-II tetramer staining and characterized. Within these repertoires, we identified public TCR sequences and motifs, validated selected TCRs for antigen specificity and affinity, and explored peptide determinants for TCR recognition.

To our knowledge, this is the first study to report the TCR repertoires responding to HHV-6B. In a previous work, we identified six major HHV-6B epitopes naturally presented by DRB1*03:01 in infected cells (48). Almost all DRB1*03:01 subjects tested had T-cell responses to the pool of these epitope peptides, but different subjects responded to a different subset of epitope peptides. In our present study, we used the same mixture of epitope peptides to expand and sort specific T cells; hence, the HHV-6B-specific repertoires reflected this subject-specific pattern of reactivity. This complexity was evidenced in the diversity measurements of the repertoires of T cells expanded with the pool compared to repertoires expanded with individual epitope peptides. Overall, our publicity assessment of HHV-6B TCR repertoires rendered a handful of public clonotypes restricted to U11- and U85-derived epitopes that were widely recognized within our population.

We also characterized TCR repertoires responding to HCMV. Several previous studies have reported TCR repertoires responding to HCMV. Most such studies focus on CD8 T cells recognizing pp65 or IE1-derived peptides, presented mainly in the context of the MHC-I alleles HLA-A*02:01 and B*07:02 (78, 90–92). Recently, TCR repertoires of CD4 T cells recognizing HCMV epitopes derived from pp65 and IE1 were reported (60, 61), although the presenting MHC-II alleles were not identified. Here, we reported TCR repertoires recognizing a well-known CD4 epitope derived from pp65 and presented by DRB1*03:01 (62, 63).

The DRB1*03:01 allele was recently defined as one of the “universally frequent alleles”, with allele frequencies above 10% in at least one population present in eight of nine defined world regions (93). This categorization places DRB1*03:01 among the more frequent DRB1 alleles and highlights the relevance of these TCRs.

The TCR repertoires of individual subjects recognizing the DRB1*03:01-restricted viral epitopes reported here were composed of a few tens to a couple hundred clonotypes with a handful of clonotypes highly expanded, both public and private. The frequency of clonotypes after *in vitro* expansion probably reflects both the *in vivo* distribution of clonotypes and their relative ability to proliferate *in vitro* in response to the antigen recall expansion (93). HHV-6B clonotypes were not detected in analyses of the total unexpanded CD4^+^ T cells from the same subjects, while some HCMV clonotypes were detected in the unexpanded samples, consistent with the relatively lower frequency of HHV-6B-specific (43, 44, 48, 49) and relatively higher frequency of HCMV-specific (28, 33, 94, 95) CD4 T cells in peripheral blood. In addition, some of the clonotypes could be tracked for over a year, indicating that these viral-specific clonotypes can persist in time. Persistence for years of CD8 clonotypes recognizing HCMV has been reported before (93, 96).

Public TCRs refer to CDR3 amino acid sequences that are shared among different subjects. These TCRs are often associated with a given antigen and hence with a disease phenotype (97), therefore the interest in studying them. We used next-generation TCR sequencing to identify TCRs recognizing HHV-6B and HCMV antigens presented by the HLA molecule DRB1*03:01. Even though the repertoires were mainly private, several public clonotypes were identified, including 25 individual CDR3α, 16 individual CDR3β, and three α/β pairs. Shared CDR3s include, along with identical clonotypes, families of highly similar clonotypes, which are believed to share the same antigen specificity. We used clustering algorithms (73, 89) to identify 67 families of related CDR3s recognizing the HHV-6B or HCMV epitopes and confirmed the shared specificity in one of our study cases.

A subset of the HHV-6B and HCMV clonotypes that we identified here was also found in previously reported datasets of non-epitope-specific TCR repertoires (74, 75), which highlights the widespread publicity of the HHV-6B and HCMV public TCRs. This overlap was more frequently observed in subjects expressing the DRB1*03:01 allele, consistent with the specificity to both viral epitope and MHC molecule. However, the presence of these clonotypes in subjects with other HLAs was noticeable. There was no overlap between any of the HHV-6B and HCMV TCR repertoires reported here with previously reported TCR sequences assigned to other epitopes, which indicates that repertoires are highly specific. For HCMV, for which TCR repertoires have been reported (14, 15, 60, 61), few public clonotypes were shared. The public CDR3α clonotypes CAANTDKLIF and CVVNQAGTALIF were shared between our dataset and data from Kar et al. (61). The gene usage was different for the first clonotype (Vα39/Jα34 vs. Vα25/Jα34) but conserved in the second (Vα12-1/Jα15), and the pairing was different in both cases (CASSPGASSYNEQFF vs. CASSVEAGSFSGELFF for the first clonotype and CATTRAGTGNTIYF vs. CAVLGGVPHEKLFF for the second). The small overlap between these datasets and ours may be a result of the different HLA restriction of the study subjects and the different approaches used to isolate the specific T cells (short vs. long *in vitro* stimulation, number and/or type of peptides used to stimulate, and sorting by activation markers vs. tetramer).

Our characterization of selected public and private TCRs showed functional avidity in the nanomolar range, which suggests relatively strong interactions (98). However, there was not a clear correlation between the functional and tetramer avidities overall. This was particularly striking for pp65.3, which had the highest functional avidity, but for which no tetramer binding was observed under the conditions tested. However, it should be noted that functional avidities were determined using a cell line expressing other MHC alleles in addition to DRB1*03:01, while tetramer staining was restricted to DRB1*03:01 only. Thus, it is possible that more than one allele could bind the added peptide, and the T-cell reactivity and measured functional avidity might reflect multiple MHC–peptide interactions.

Identification of peptide residues important for TCR recognition gave insight into the nature of pMHC–TCR interactions involved. For the pp65.3 TCR, only seven residues of the peptide were required for T-cell recognition, all of them within the predicted 9mer binding core. This contrasted with the nine residues required for T-cell recognition by pp65.0, which includes the p−1 position of the predicted binding core. Both pp65 TCRs have the same Jα usage and share the AAN motif; both CDR3β had different Vβ and Jβ usage, but the common motif (R/P)GA(G/S) may be important for the pMHC recognition. In the original paper describing this epitope, the reactivity was associated with subjects expressing alleles DR3 (DR17) and DR52 (62), and in Bronke et al. (63), the DRB1*03:01 tetramer reactivity was not totally correlated with IFNγ production, suggesting that this epitope may be presented by more than one allele. Peptide binding predictions of pp65.509–523 to DRB1*03:01 (DR3) and DRB3*01:01 (DR52) showed a stronger predicted binding to DRB3*01:01. Thus, it is possible that both alleles were able to stimulate the CD69 expression, and lower binding for DRB1*03:01 was responsible for the lack of tetramer recognition in our transduced cells. This TCR was very abundant in the *in vitro* expanded T-cell lines, which could be better recognized by the low-affinity tetramer.

The two U11-specific TCRs apparently use unrelated CDR3 motifs recognizing the same pMHC. This highlights plasticity in the pMHC–TCR interaction, in which different residues in the CDR3 can interact with different residues in the peptide and in the MHC, along with other regions outside the CDR3, maybe including CDR1 and CDR2 (99, 100). Both public U11 TCRs had very different CDR3α and CDR3β sequences, with different Vα, Jα, and Vβ usage and only sharing Jβ usage. Both U11 TCRs were activated specifically by the U11 peptide and bound the specific tetramer, although with differences in functional avidity and tetramer avidity. The minimal peptide necessary for TCR recognition was the same for both TCRs, which suggests that each TCR probably established a unique set of interactions with the peptide residues available.

The two U85-specific TCRs were an example of highly similar TCRs for which we confirmed the same antigen specificity. Both clustered together after analyzing the data using the clustering algorithms (73, 89). They shared a similar motif on the CDR3α, with the same usage in Vα, Jα, and Vβ, but different usages for Jβ. Both TCRs were activated specifically by the U85 peptide, had similar functional avidities, and bound the tetramer, although there were differences in tetramer avidities. The (S/T)NS motif in the CDR3α of both TCRs could be accommodated in the same manner, but, in the CDR3β motif R(V/G)G, the V/G change may affect the interaction and explain the differences in tetramer avidity.

Both HHV-6B and HCMV are viruses that establish lifelong latent with reactivation events (101–103). They can be reactivated in immunocompromised individuals and individuals under T-cell immunosuppression after solid organ or hematopoietic stem cell transplantation [reviewed in (104, 105)]. In transplant settings, different approaches have been proposed and used to control reactivated viruses, including the restorative transfer of virus-specific expanded T cells, genetically engineered T cells expressing virus-specific transgenic TCRs, and engineered TCRs (106–113). *In vitro* expanded T cells have been proposed as a therapeutic measure to control viral reactivation, including HHV-6B and HCMV, after transplantation (105, 107). Our gene expression profiles of the HHV-6B and HCMV *in vitro* expanded CD4 T cells revealed a cytotoxic phenotype in both cases. However, differentially regulated genes in HHV-6B- and HCMV-expanded cells suggest functional differences. The HCMV-expanded CD4 T-cell gene expression landscape suggests a robust cytotoxic profile. On the other hand, the HHV-6B-expanded CD4 T-cell gene expression landscape still supports an increased cytotoxic capacity compared to total unstimulated CD4 T cells, but less than that of HCMV-expanded cells. These observations are consistent with the reduced specific lysis by HHV-6B-expanded T cells as compared to HCMV-expanded T cells observed in functional assays.

Immune control of HCMV has been associated with both CD8 and CD4 T cells, where CD4 T cells are necessary for a more rapid and positive outcome [reviewed in (58)]. HCMV-specific CD4 T cells with cytolytic capabilities have been previously described (32, 33, 60, 61, 94, 114). Functional differences between HHV-6B and HCMV responding T cells have been previously reported, including lower frequencies of HHV-6-responding T cells, a central-memory phenotype in the effector cells, and higher frequencies of regulatory T cells (87). Most of these results are consistent with our findings, and differences in finding a clear regulatory T-cell phenotype may be derived from different experimental approaches used, like length of the *in vitro* stimulation.

## 5 Limitations

There are limitations to our work. To better understand the relevant publicity associated with a given antigen, the abundance of the clonotypes in the subjects should be considered, but our analysis did not account for that factor. We only assigned TCR clonotypes to three epitopes, although responses to six epitopes were observed. TCR clonotypes were investigated only in six subjects for HHV-6B and four subjects for HCMV, and additional clonotypes including other public ones would likely be observed in a larger cohort. It is possible that our observation of more functional heterogeneity in HHV-6B-expanded cells as compared to HCMV-expanded cells could be because they are recognizing six different HHV-6B epitopes as compared to a single HCMV epitope, but we did not investigate the functional profile of individual epitopes. We observed lower frequencies of HCMV^+^ CD4 T cells in our *ex vivo* data than that reported by others, which may be the result of using a single epitope as compared to more complex sources of antigens like pools of peptides or infected cell lysates. Indeed, Sylwester et al. (28) reported ~10% of the T cells as HCMV-reactive, with these frequencies corresponding to the average of the combined response to several pools of overlapping peptides covering the whole virus proteome in multiple subjects. In previous work, we described a similar situation, in which we observed better responses to a pool of peptides than to individual ones (48). Lastly, the Vavy cell line used to present epitope peptides in the functional assays expresses three other MHC-II proteins in addition to DRB1*03:01: DRB3*01:01, DQA1*05:01/DQB1*02:01, and DPA1*02:01/DPB1*01:01, and it is possible that some of the observed T-cell responses involve peptides presented by these proteins.

## 6 Conclusions

We report here the characterization of TCR repertoires recognizing previously reported immunodominant epitopes from two widely spread viruses that latently infect human populations, and which can cause problems in immunocompromised patients. The importance of the subset of CD4 T cells with cytotoxic capacity has been increasingly recognized, especially in the context of chronic viral infections but also in other settings like transplantation and cancer (115–117). We verified the cytotoxic capacity of CD4 T cells recognizing immunodominant epitopes from HHV-6B and HCMV and characterized their TCR repertoires using peripheral blood samples from six subjects. Within these repertories, we identified many public TCR clonotypes shared across donors. These include 41 individual CDR3α or CDR3β and three TCRα/β pairs. In many cases, families of related sequences presumably recognizing the same epitope were also observed. We identified these TCRs in antigen-specific T-cell populations expanded *in vitro*, but these clonotypes could be detected in reported datasets of total peripheral blood without expansion. Six TCRα/β pairs were validated in studies of transduced T cells, including both public and private clonotypes recognizing HHV-6B U11, HHV-6B U85, and HCMV pp65 epitopes. Transduced cells specifically recognized peptide-pulsed targets with high avidity, with half-maximal peptide concentrations ~5–50 nM. Core epitopes were identified in each case. The HHV-6B and HCMV public clonotypes reported here can be useful as part of diagnostic or therapeutic strategies, by tracking infection, reactivation, and response to therapy.

## Data Availability

The data presented in the study are available in the ArrayExpress database (http://www.ebi.ac.uk/arrayexpress) under accession numbers E-MTAB-16144 and E-MTAB-16174. Epitope data are reported in the IEDB with accession numbers 921620 (U11), 872484 (U56), 920761 (U85), 34578 (pp65); the corresponding TCRs are under Reference ID 1047549.
