# Supplementary material for: TCR repertoire of human cytotoxic CD4 T cells responding to betaherpesviruses HHV-6B and HCMV
Source: Front Immunol. 2025 Nov 25;16:1631558. doi: 10.3389/fimmu.2025.1631558 (PMC12687561; doi:10.3389/fimmu.2025.1631558)
Supplement: Supplementary file 8 [file DataSheet1.pdf]

Supplementary Material

1 Supplementary Figures

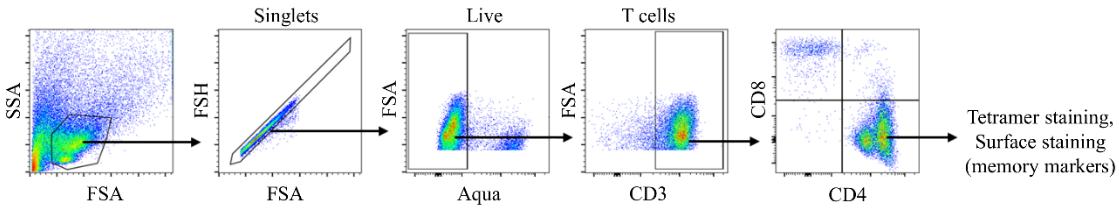

Figure S1. Gating strategy for flow cytometry analysis of T cells. Sequential selection using size and granularity (FSA and SSA), singlets, live cells, CD3, and CD4 and CD8 expression. Representative dot plots for in vitro expanded T cells. The CD4+CD8- population was used for assessing tetramer staining and activation markers.

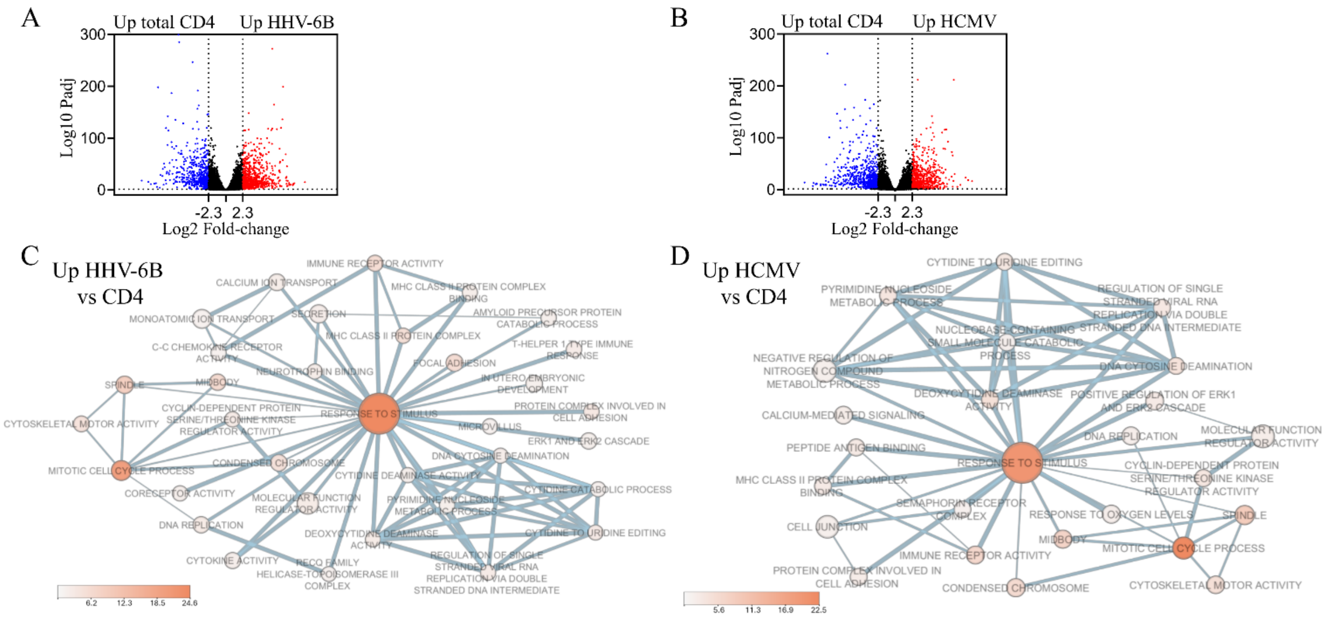

Figure S2. Differential gene expression analysis on total unexpanded CD4 T cells and HHV-6B or HCMV-expanded and tetramer-sorted CD4 T cells by bulk RNA sequencing. **(A).** Volcano plot representation of differentially regulated genes; in color are shown the statistically significant genes upregulated at least 5-fold in total CD4 T cells (blue) or HHV-6B T cells (red), Padj <0.05. **(B).** Same as **(A)** but for HCMV. **(C).** Functional profile network for genes upregulated 5-fold or more in HHV-6B-expanded cells compared to total CD4 T cells. **(D).** Same as **(C)** but for HCMV. For all networks, node fill color= -Log10(P-value).

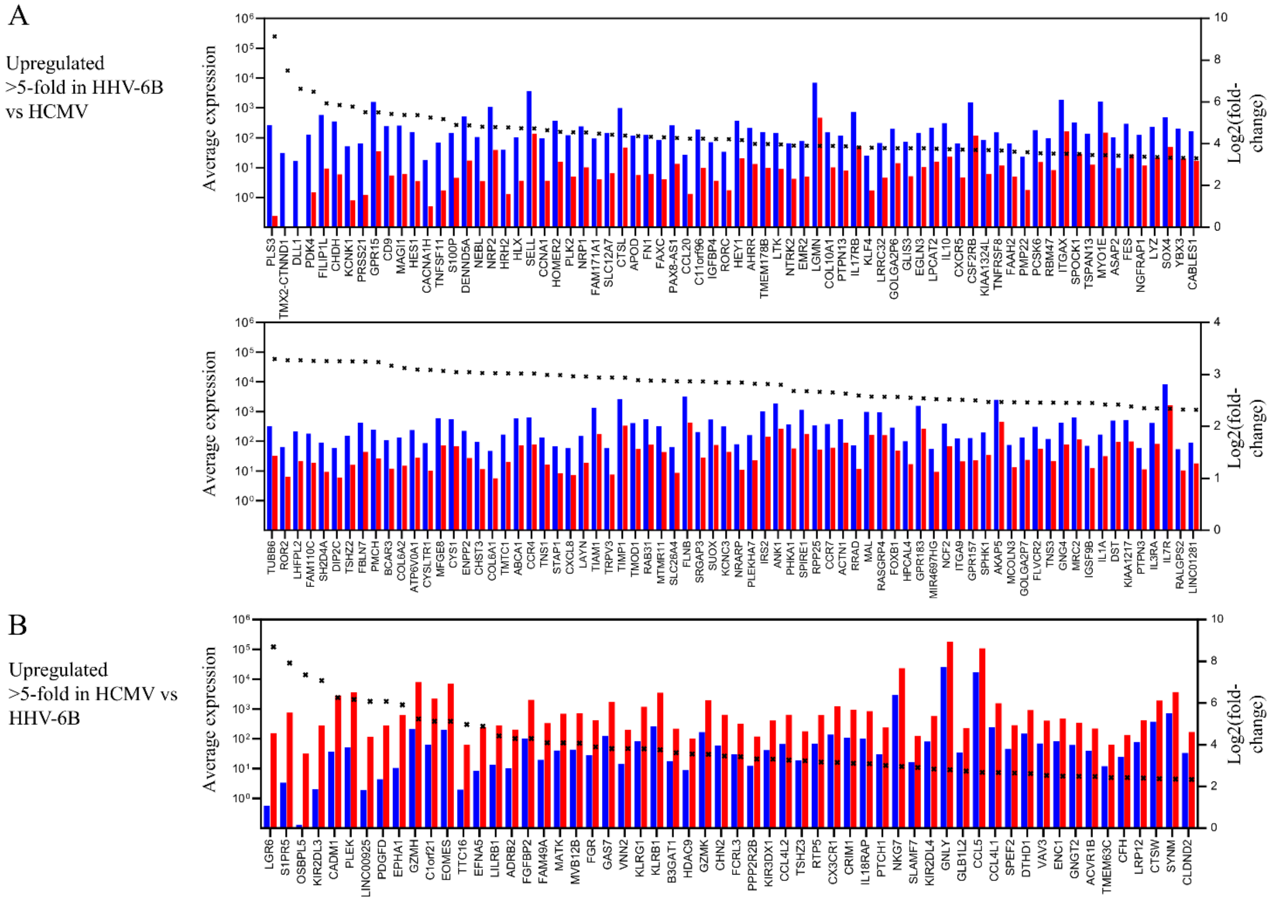

Figure S3. Average expression levels (CPM) of genes differentially expressed 5-fold or more when compared HHV-6B vs HCMV in vitro-expanded and tetramer-sorted T cells (bulk RNA sequencing). **(A).** Upregulated in HHV-6B. **(B).** Upregulated in HCMV.

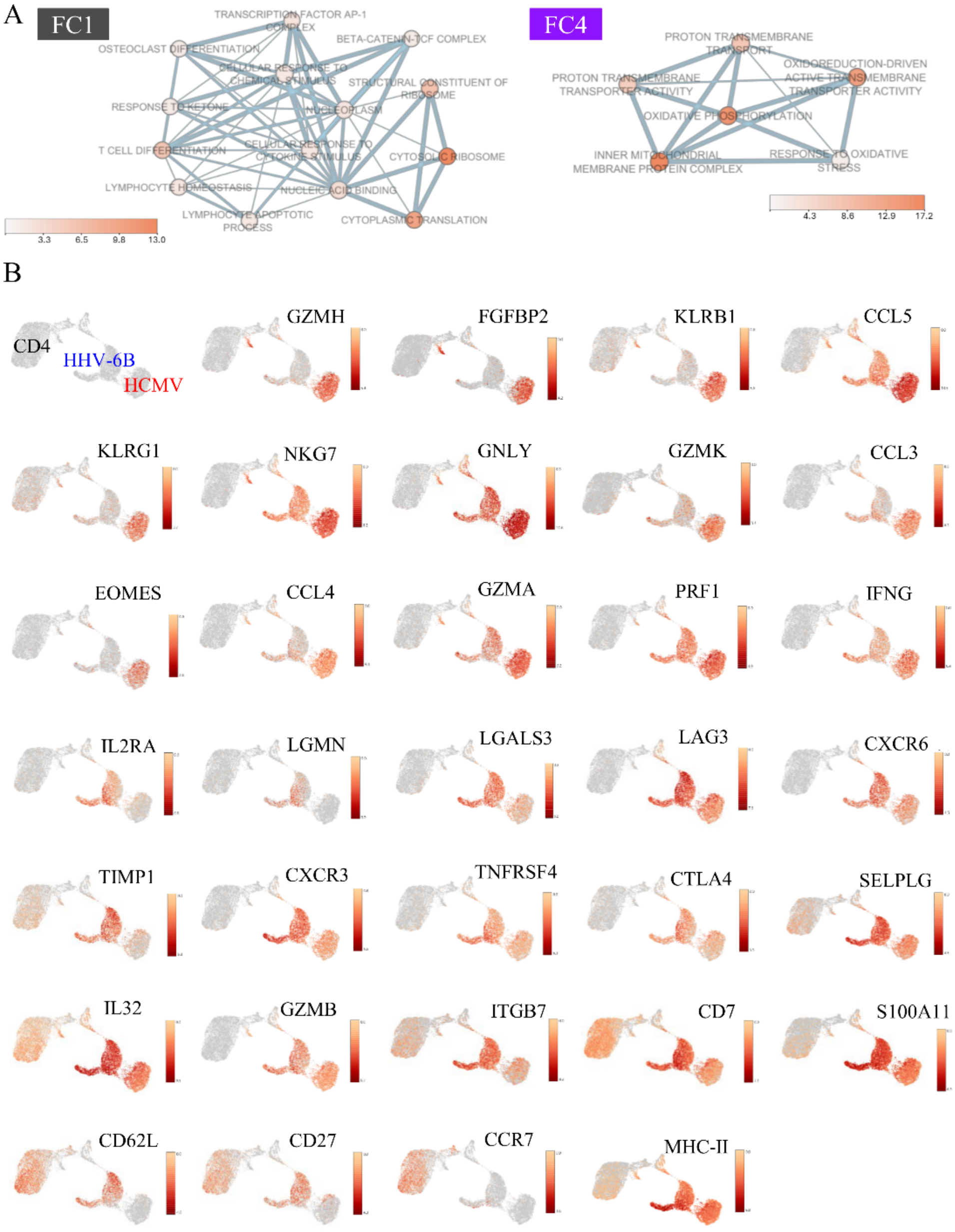

Figure S4. Single-cell RNA sequencing. **(A).** Functional profile network for genes upregulated in clusters 1 and 4 (FC1 and FC4) (node fill color= -log10(p-value)). For all networks, node fill color= -log10(p-value). **(B).** UMAP projection of selected differentially expressed genes on single-cell RNA sequencing. The top plot shows the location of each population. Expression levels of each gene in each subpopulation given by the bar next to each plot.

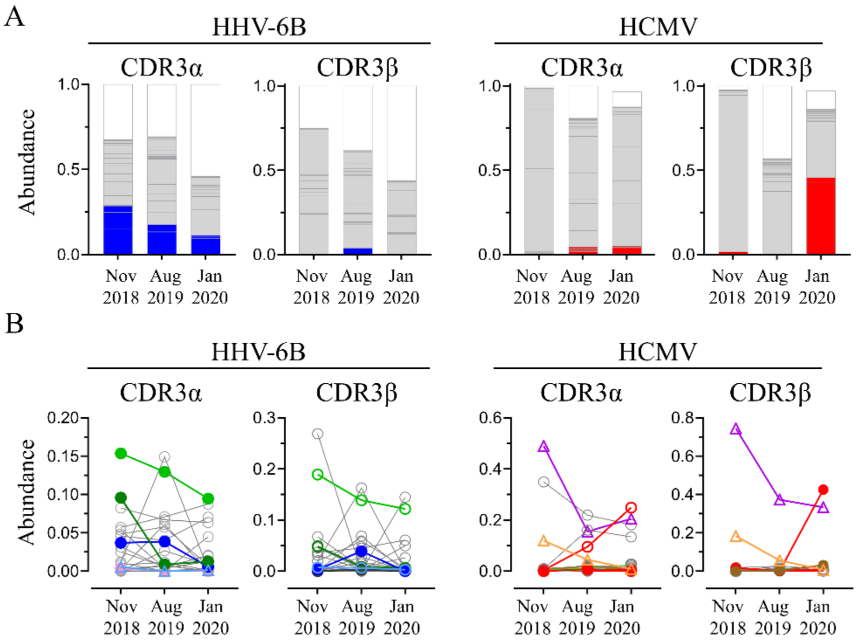

Figure S5. Tracking clonotypes identified in d368. **(A).** Fractional abundance of HHV-6B or HCMV clonotypes that match at the amino acid level in samples from the same subject collected at different times (Nov 2018, Aug 2019, Jan 2020); public clonotypes are shown in blue for HHV-6B and red for HCMV; other clonotypes present at different times are shown in grey; and clonotypes unique for each timepoint are shown in white. **(B).** Public (solid circles) and highly-similar clonotypes (open circles) present in different samples. The following selected clonotypes are shown in color. For α chains: CVVNIFTGNQFYF (dark blue); CAASNSGNTPLVF (light green); CAATNSGNTPLVF (dark green); CASSGAGSYQLTF (light blue); CAVNSGAGSYQLTF (pink); CAVNTDKLIF, CAANTDKLIF, CAASTDKLIF, CAGSTDKLIF (red); CAVRKGGNTPLVF (gold); CAVDKARLMF (turquois); CAVTPGNQFYF (emerald). For β chains: CASSLTTAGEEKLFF (light blue); CASSFRGGEKLFF (dark blue); CGNRVGNSPLHF (light green); CSARRGGGPQHF (dark green); CASSPGASSYNEQFF, CASSPGSSSYNEQFF, CASSPGTSSYNEQFF (red); CASSLGRSSYNEQFF (brown). Private clonotypes are shown as open triangles: CGAANTDKLIF and CSARGAGMSYTF (α and β, orange), CAVKKAGNTPLVF and CSPGGGTYEQYF (α and β, purple).

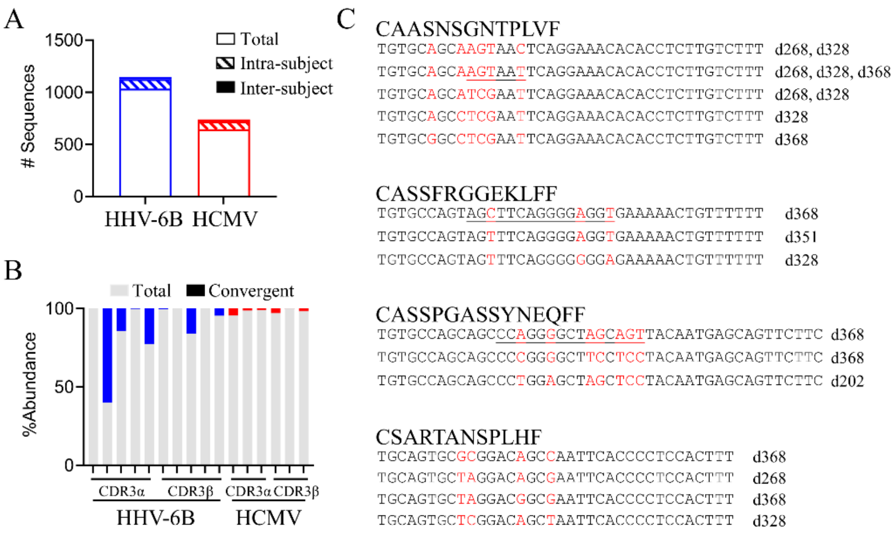

Figure S6. CDR3 sequence convergence. **(A).** Number of convergent sequences observed in HHV-6B (blue) and HCMV (red) clonotypes; bars represent all CDR3α and CDR3β amino acid sequences, with the number of intra-subject convergent sequences shown as diagonal pattern, and the number of inter-subject convergent sequences (public) as a solid fill. **(B).** % Abundance of convergent sequences (% of total counts, solid fill) for HHV-6B (blue) and HCMV (red) clonotypes; each bar represents one subject. **(C).** Examples of the intra and inter-subject sequence convergence observed in our data; on top the amino acid sequence of the clonotype, followed by a list of the different nucleotide sequences encoding it and the subjects where they were observed. The V(D)J junction is shown in the sequence used for cloning.

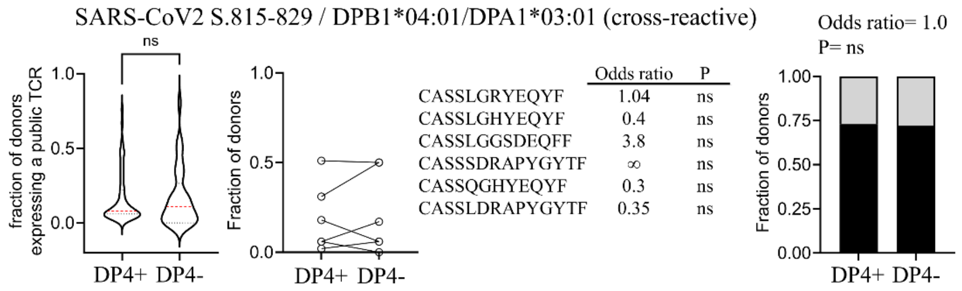

Figure S7. Publicity of CDR3β of cross-reactive clonotypes recognizing SARS-CoV2 S.815-829 presented by DPA1*01:03/DPB1*04:01 (65) in datasets of unsorted TCRs (similar analysis as in Figure 5).

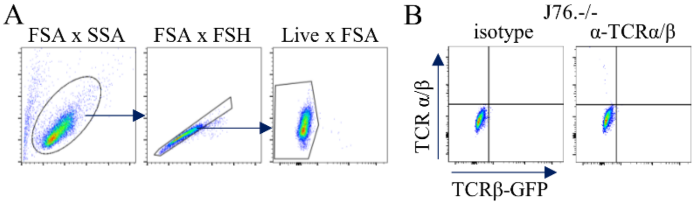

Figure S8. Analysis of single-TCR transduced cells. **(A).** Flow cytometry gating strategy. **(B).** Representative dot plot of non-transduced J76.-/-.CD4.Luc cells stained with IP26 (BV421 for TCRα/β and GFP for TCRβ expression).

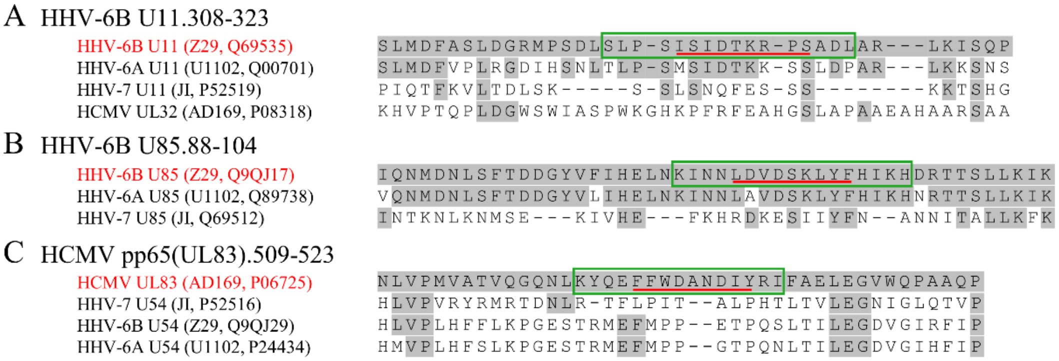

Figure S9. Positional homology of HHV-6B and HCMV epitopes in other betaherpesviruses. Clustal Omega Multiple Sequence Alignment (EMBL-EBI) of **(A)** U11 from HHV-6A, HHV-6B, HHV-7, and HCMV UL32; **(B)** U85 from HHV-6A, HHV-6B, and HHV-7; and **(C)** HCMV pp65 (UL83) and U54 from HHV-6B, HHV-6A, and HHV-7. Identical residues in each position of the alignment are shaded in grey. The sequences of the peptides (Table 1) corresponding to the HHV-6B U11.308-323 and HHV-6B U85.88-104, and for the HCMV pp65.509-523 are boxed in green, and the predicted 9mer binding core (NetMHCIIPan, v4.3) underlined in red. In all cases, the virus strain and the protein accession number are shown in parenthesis.

2 Supplementary Tables Captions

Table S1: Oligonucleotide sequences used for bulk TCR sequencing.

Table S2: Differentially expressed genes uncovered by bulk RNA sequencing. **(A).** Total CD4 vs HHV-6B. **(B).** Total CD4 vs HCMV **(C).** Total HHV-6B vs HCMV. Expression levels for each group (CPM), Padj and Log2(Fold-change).

Table S3: Differentially expressed genes uncovered by single-cell RNA sequencing. Average expression levels in each cluster, Log2(Fold-change), and P-value (globally distinguished gene expression data). **(A).** Functional cluster (FC) 1. **(B).** FC2. **(C).** FC3. **(D).** FC4. **(E).** FC5.

Table S4: CDR3 sequences. **(A).** Summary of CDR3α and CDR3β identified by bulk sequencing for each subject and antigen (total counts, number of clonotypes, and frequency of the top-5 clonotypes). **(B).** Summary of TCRα/β pairs identified by single-cell TCR sequencing for each subject and antigen (total counts, number of clonotypes, and frequency of the top-5 clonotypes). **(C).** List of CDR3 clonotypes identified by bulk TCR sequencing. **(D).** List of TCRα/β pairs identified by single-cell sequencing.

Table S5: Public HHV-6B and HCMV CDR3β clonotypes found in other datasets of TCRs derived from unsorted PBMCs and CD4 T cells. **(A).** HHV-6B; **(B).** HCMV. The number of subjects in the DRB1*03:01 positive and negative populations in which a clonotype was found and the total number of subjects in each group are shown. Highlighted are the public clonotypes in our dataset (only). Similar analysis is shown for clonotypes recognizing IAV M1.58-66 **(C)** and HCMV pp65.495-503 **(D)** presented by A*02:01; and two epitopes of SARS-CoV2 (S.167-180 **(E)** and S.815-829 **(F)**) presented by DPA1*01:03/DPB1*04:01.

Table S6: GLIPH2 convergence groups in HHV-6B and HCMV CDR3β. **(A).** HHV-6B. **(B).** HCMV.

Table S7: Predicted cross-reactivity of TCRs. [1] Binding predictions for each peptide and their variants in other viruses were obtained using NetMHCIIPan (v4.3) for DRB1*03:01 and DRB3*01:01. [2] Predicted binding core. [3] Prediction rank. [4] TCR recognition score as follows: a. Align the binding cores of the study peptides to the peptide that is a known epitope for a given TCR. b. Assign weighted values for residue identity at each peptide position: p1= +1, p2= +3, p3= +2, p4= +1, p5= +5, p6= +1, p7= 2, p8= +3, p9= +1. c. Calculate the score as fraction of the known epitope. A value closer to 1 indicates a better chance of cross-reactivity.
